# “It’s where I buy lunch or anything that I want to buy”: a participatory photo elicitation study on adolescents’ experience of food choice and environment in Kisumu, Kenya

**DOI:** 10.1186/s41043-026-01421-8

**Published:** 2026-08-24

**Authors:** Elisa Gobbo, Alice Achieng Ojwang, Kristi Sidney Annerstedt, Joseph Ochieng Amoke, Loyce Joyce Anyango, Sibylle Herzig van Wees, Donvan Nunda, Edward Ouko, Esther Oruko, Helle Mølsted Alvesson

**Affiliations:** 1 Department of Global Public Health, Karolinska Institutet, Tomtebodavägen 18, Solna, 171 65 Sweden; 2 Department of Health and Biomedical Sciences, The Technical University of Kenya, Haile Selassi Avenue Entrance on Workshop Road, PR5G+F4, Nairobi, Kenya; 3 Department of Agriculture, Irrigation, Livestock, Fisheries, and Blue Economy, County Government of Kisumu, Prosperity House, VQR2+86, Kisumu, Kenya

**Keywords:** Food environment, Food choice, Adolescents, Photo elicitation, Kenya, Qualitative, Informal settlements, Participatory research

## Abstract

**Background:**

In Kenya, adolescents face a rapidly changing nutritional landscape, a growing triple burden of nutrition, and a rising risk of non-communicable diseases. Behaviors that begin in adolescence can influence lifelong dietary patterns and future disease risk. Yet, there is limited understanding of contextually grounded, adolescent-centered experiences of their food environments and food choice in the African Region. This study aims to address this gap by exploring how young adolescents attending school in an informal settlement in Kisumu, Kenya, experience and visualize their local food environment.

**Methods:**

This is a qualitative participatory study using the visual methods of photo elicitation and social foodscape mapping. Participants took photos of their food environment and then participated in group-based discussions and mapping with researchers. The participants were recruited using convenience sampling from grade 7 until information power was reached. Eighteen adolescents participated (9 females, 9 males, ages 12–13 years). Nine have access to a phone at home. 157 unique images and 17 map drawings were created. Data were analyzed using Drew & Guillemin’s three-stage methodology and an abductive reflexive thematic analysis to interpret both images and narratives.

**Results:**

The analysis generated three main themes: (1) food choices are shaped by convenience and familiarity, yet the adolescents express an interest in items beyond the familiar, (2) adolescents mostly receive food from adults but are beginning to make some independent choices, and (3) adolescents value the social dimensions of food, yet they navigate different forms of belonging across family and peer contexts. Overall, adult routines and provisions remain central, but adolescents value moments of autonomy and express curiosity for more processed and peer-valued foods.

**Conclusions:**

These young adolescents in Kisumu are navigating an early transition point shaped by financial constraints, physical accessibility, and adult-led routines. However, they also express pride in independent choice and interest in greater influence. This signals they are developing volitional autonomy within their social and environmental constraints. These findings position early adolescence as a critical window for intervention to enhance adolescents’ capacity to negotiate healthy food choices and influence lifelong dietary patterns.

**Supplementary Information:**

The online version contains supplementary material available at 10.1186/s41043-026-01421-8.

## Background

Early adolescence marks a critical period in the life-course when individuals begin transitioning and interacting with their food environment in ways distinct from adults [1, 2]. This change is not merely a logical response to the environment; rather, it reflects negotiated decisions embedded in social norms, cultural meanings, and structural constraints [3–5]. Globally, the early adolescent stage, defined as young adolescents between the ages of 10 to 14, represents a turning point when they are learning to negotiate complex factors and form habits that can have life-long implications for their nutrition and health [1, 6, 7].

**Fig. 1:**
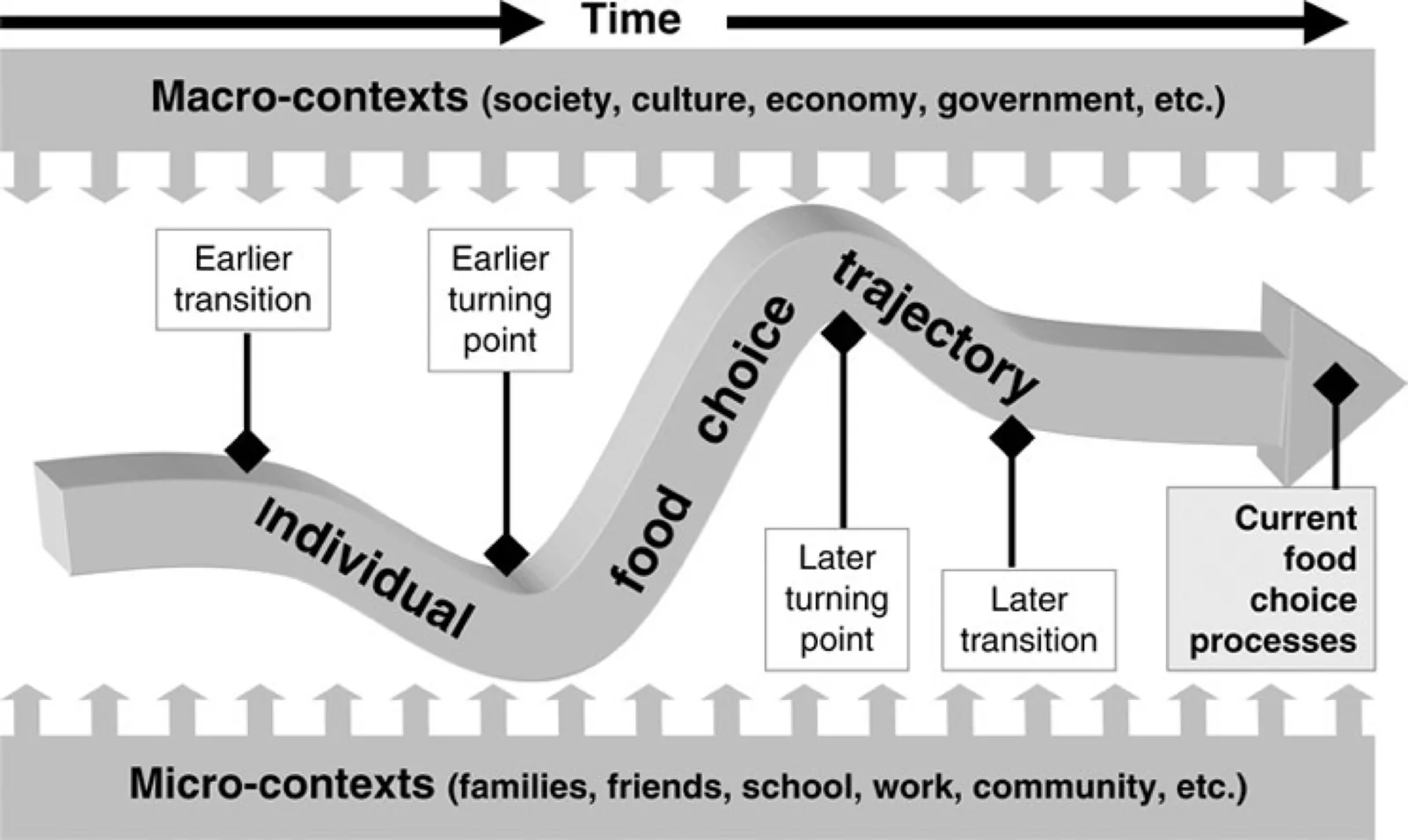
Conceptual model of food choice over the life course (from Sobal et al. [8])

Drawing on Sobal’s conceptual model (Fig. 1), we frame food choice decision making as a dynamic and context-dependent process that is’ shaped by various values and influences [8]. The model distinguishes between macro-contexts (political, economic, cultural, societal, or governmental) and micro-contexts (family, peers, school, or community) through which adolescents encounter and interpret their food environment [8]. Within these contexts, attributes such as availability, affordability, convenience, and opportunity influence or constrain interactions within their food environment [9, 10]. Through negotiations in their context, adolescents form food choice trajectories that evolve, potentially consolidating or redirecting dietary patterns into adulthood [8].

We position young adolescents at an “early transition” stage in the model, where their food choices are shaped by both micro- and macro-contexts. During this early transition, adolescents begin to develop autonomy, understood as self-endorsed and volitional functioning [11]. Particularly relevant in collectivist contexts, volitional autonomy de-emphasizes individualistic ideas of independence and rather prioritizes the quality of personal motivation and the presence of supportive relationships [11]. As adolescents grow up and their contexts evolve, they are learning to navigate shifting influences that begin during this formative period. The nutritional transition underway in many African settings is reshaping adolescent diets, with significant implications for health across the life course [6, 12, 13]. As traditional, minimally processed diets yield to energy-dense, ultra -processed foods, adolescents face heightened vulnerability to malnutrition in its multiple forms [6, 12, 13]. This shift contributes to the “triple burden” of malnutrition characterized by persistent undernutrition and micronutrient deficiencies alongside rising rates of overweight and obesity, which ultimately amplifies the adolescents' future risk of non-communicable diseases (NCDs) [14–16].

Against this backdrop, understanding young adolescents’ engagement with their food environment is essential for elucidating future food choice trajectories in rapidly evolving landscapes. Existing studies highlight how adolescents’ food choices are heavily influenced by convenience, cost, availability, and peer behavior [17–19]. However, most evidence comes from quantitative data or high-income settings, leaving out important cultural and adolescent-centered perspectives [17, 20–22]. Emerging qualitative research across the African and Southeast Asian regions [23] suggests that adolescents perceive their food environments as dominated by exciting, socially desirable, cheap, and ultra-processed foods, whereas fruits and healthier options are considered expensive and monotonous [19, 24–27]. Specifically, community-based studies from Nairobi found that social factors, affordability, local food availability, and time constraints are major drivers of food choice [27–29].

Despite existing insights, research rarely centers on younger adolescents’ voices and experiences in African informal urban settings. To address this gap, our study employs an innovative participatory methodology that utilizes photo elicitation and foodscape mapping to amplify adolescents’ perspectives. This approach provides contextually grounded insights into how young adolescents negotiate emerging volitional autonomy within their food environment and advances the conceptualization of adolescents’ early food choice transitions. Specifically, we aim to understand how young urban adolescents experience and visualize their local food environment in an informal settlement in Kisumu, Kenya.

## Methodology

### Study design

The study uses a qualitative participatory research design approach involving the visual methods of photo elicitation and social foodscape mapping. The methods were inspired by photovoice and foodscape mapping walk-abouts [30–32]. Both methods served to generate insights into young adolescents’ foodscapes, defined as the food environment and ways it is shaped by social practices, institutions, and power relations [32, 33]. In March 2025, we actively engaged seventh-grade adolescents (ages 12–13 years) in documenting their food environment through photography, map drawing, and reflections.

This study lies within a constructivist paradigm [34, 35]. Our epistemological stance values adolescents as experts in their own lives and produces knowledge collaboratively through co-construction between adolescents and the research team [34, 35]. Specifically, the methods drew on the foundations of photovoice: Freire’s emphasis on experiential knowledge, critical feminist theory’s focus on amplifying marginalized voices, and photography as a tool for consciousness-raising [35, 36]. The visual materials from photos and maps served to center younger adolescents’ perspectives and stimulate discussion and reflection on paths for improving healthy environments and behaviors [31].

### Setting

This study is embedded within the EU Horizon Changemaker project, which aims to co-design, implement, and evaluate a school-based sustainable health intervention on health, nutrition, and environmental outcomes for the primary prevention of NCDs related to the triple burden of nutrition with adolescents in three rapidly urbanizing cities in Burkina Faso, Kenya, Tanzania [37]. The intervention involves urban farming, sustainable health modules, health conversations using motivational strategies, and mass media campaigns. This study is conducted within the project’s co-design phase to help inform the adaptation of the intervention components in Kenya. All data collection was completed in one of the Changemaker intervention schools prior to implementation [36]. 

The study was conducted in an urban informal settlement area in Kisumu, Kenya’s third largest city, located along Lake Victoria [38, 39]. Kisumu is a rapidly urbanizing city, with 47% of its population living in informal settlements characterized by poverty, insecure housing, overcrowding, limited services, and food insecurity [38, 39]. The population is characterized by high unemployment (up to 36%), a largely informal workforce (nearly 70%), and a predominantly young population (over half under the age of 20) [38–40]. Kisumu’s food environment is dominated by markets and informal food vendors. Around 70% of food vendors operate in licensed marketplaces or shops, while approximately 19–30% trade informally [41]. These informal food vendors are vital in informal settlements, offering more affordable and accessible foods, sometimes on loan or in smaller quantities [41]. Recent data from Kisumu illustrates the existence of the triple burden of malnutrition, exemplified by approximately 9% under-five stunting, overweight and obesity rates of 7.9% and 14.4%, respectively, and deficiencies in iron, vitamin A, vitamin D, and zinc among children and adolescents [42–45].

The study was conducted in a comprehensive junior secondary school designated as a Changemaker intervention school and engaged in the project’s co-design phase. Many adolescent students access lunch through a non-governmental program that funds and prepares meals in centralized kitchens and distributes them to school stalls, where the adolescents pay a subsidized amount of 15 shillings (0.10€) per day via a digital wristband system preloaded by their guardians [46]. A few adolescents carry lunch from home if they cannot afford the program.

### Participants and recruitment

Recruitment followed convenience sampling based on school-suggested procedures to recruit via teachers and emphasized adolescent choice. In coordination with the Vice Principal and teachers, 20 seventh-grade adolescents were recruited. The intended strategy was a purposive sampling based on gender balance, varied proximity to school, and socioeconomic diversity, but as the teachers’ selection could not be verified, the final sample functioned as a convenience sample. The teachers distributed study information, guardian consent, and adolescent assent forms for them to review and take home. Adolescents were therefore able to decide whether to take the information and discuss participation with their guardian before providing their own assent. An initial meeting with recruited adolescents and interested guardians provided detailed study information and addressed questions verbally. Adolescents received refreshments of water and an apple or yogurt during data collection and a folder with two notebooks as compensation for the time spent with the research team.

Although all 20 adolescents returned signed consent and assent forms, one was withdrawn by a parent, and one did not attend any meeting despite three invitations, resulting in a final sample of 18 adolescents with nine females and nine males. They were all from Grade 7; ten were 12 years-old and eight were 13 years-old. Half of the adolescents (*n* = 9) had access to a phone at home, but none had their own phone.

The adolescents were organized into four single-gender groups, with sessions conducted by facilitators of the same gender. Each group met with the research team twice, with 10 adolescents participating during the first week (Groups A (girls) and B (boys)) and eight (Groups C (girls) and D (boys)) participating the following week. During data generation, each adolescent took at least five photos, resulting in 157 unique images, and each produced a map drawing for a total of 17 maps (two participants collaborated on one map). The sample size was guided by the concept of information power that considered sample specificity, data quality, and analysis strategy; thus, 18 adolescents were deemed sufficient to generate meaningful insights from the photos, foodscape mapping, and group discussions [47]. The participant demographics are shown in Table 1.

**Table 1:** Demographic characteristics of the participants

Characteristics	Participants (*n* = 18)
Sex	
Female	9
Male	9
Age	
12	10
13	8
Grade in School	
Grade 7	18
Able to access a phone at home	
Yes	9
No	8
No Response	1

### Data collection

**Fig. 2:**
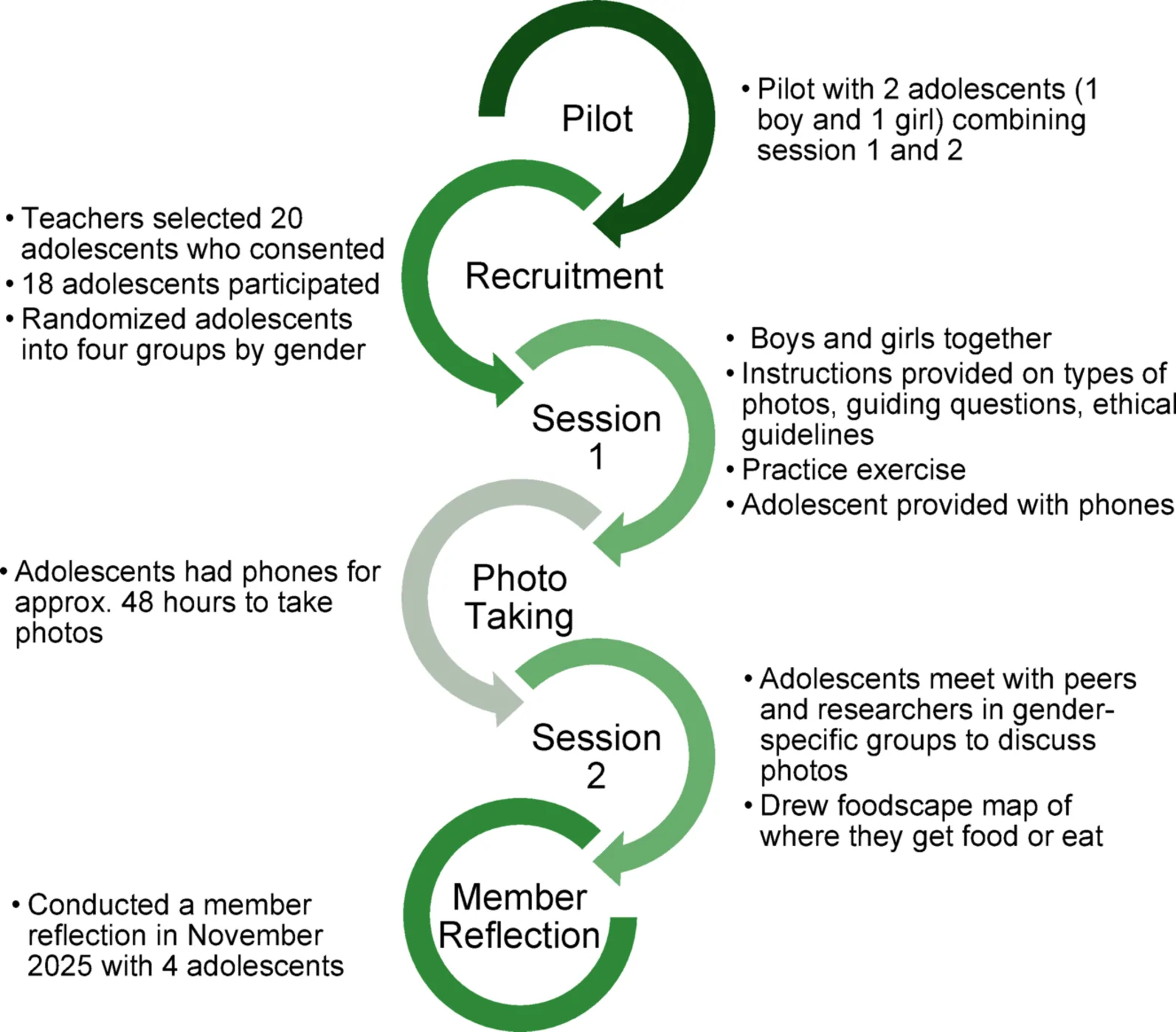
Diagram of the data collection process

The data collection followed the process of pilot, recruitment, a learning session (session 1), adolescent photo taking, and group discussion with foodscape map drawing sessions (session 2) (Fig. 2). A pilot was conducted with two adolescents from a different school within the area to refine the procedures and tools before the main data collection. The facilitators included four researchers; all in their 20 s, with two from Kisumu who speak English, Kiswahili, and Luo, one from Nairobi who speaks both English and Kiswahili, and the main author from Sweden who speaks English. All facilitators received training on qualitative data collection skills.

In the first session, facilitators provided the adolescents with instructions on types of photos, guiding questions on what to photograph, ethical guidelines, and a practice exercise (Supplementary File 1). These sessions were done with girl and boy groups together. Then, over the subsequent two days (Thursday to Saturday), the adolescents were given simple digital phones to photograph where, what, when, and with whom they eat or drink.

In the second session, the adolescents met with the facilitators and completed demographic forms, shared and engaged in group discussions about the meaning of their photos, and mapped their foodscapes. The participants described their food environment and mapped where they typically get food, and took pictures. This was guided by a storyboard with suggested processes and guiding questions (Supplementary File 2). Data collection was performed mostly in English, but with Kiswahili or Luo used as needed. Sessions took place in school classrooms, with session one lasting about 45–60 min and session two lasting approximately 90 min. Each adolescent took at least five photos, resulting in 157 unique images, and each produced a map drawing for a total of 17 maps (two participants collaborated on one map). Both sessions were audio-recorded with verbal consent. The procedures were repeated the following week with the second two groups of adolescents. To enhance the findings, a member reflection was conducted after the initial phases of analysis. Two facilitators met with four of the prior participants (two girls and two boys) to review, provide reflections, and elaborate on selected photos and simplified versions of the themes (Supplementary File 3).

### Data analysis

The analysis drew on the photos, map drawings, group discussion transcripts, and observation notes. Audio recordings were transcribed with Microsoft 365 and Otter.ai and then translated as necessary. The analysis followed Drew & Guillemin’s inductive three-stage approach: (i) adolescent-driven meaning-making from discussions on their initial interpretations of the photos and maps, (ii) researcher-driven meaning-making, combining abductive reflexive thematic analysis with question-guided inductive photo analysis, and (iii) re-contextualization where emergent themes were interpreted within theories and frameworks and the wider context [48–50]. Stages two and three were iterative and involved coding, categorizing, mind mapping, theme refinement, and recontextualization with wider theories and frameworks, all supported by Nvivo 14 [51]. Coding and analysis were driven by the first author and involved guidance from the senior author and regular discussions with the rest of the research team to improve methodological and contextual insights. Insights were also informed by the authors’ involvement in the Changemaker project, including context assessment activities, concurrent co-design data collection, and ongoing input from consortium members familiar with the schools and adolescents.

### Reflexivity and positionality

The research team involved a collaborative group of researchers across disciplines and contexts, including Kisumu, Nairobi, and Sweden. The teams have been working closely since Jan 2024. The first author, a doctoral student at Karolinska Institutet, originally from the US, had prior experience in Kisumu and spent a month in the study setting, and collaborated closely with the Kenyan partners for data collection. Data collection and initial analysis from debriefing were completed by the first author alongside Kenyan partners, followed by initial coding by the first author. The analysis was then refined collaboratively, with Kenyan partners providing key contextual insights to balance insider and outsider perspectives.

The team members included researchers from the Technical University of Kenya in Nairobi, the Kisumu County Government, and Karolinska Institutet. The team held combined expertise in social sciences, public health, nutrition, community health, and adolescent research experience. To mitigate power dynamics with the adolescents, the team used a mix of languages (English, Luo, and Kiswahili), participated in school events, and applied icebreakers and active listening. The main author maintained a reflexive journal and engaged in continuous dialogue with the team to examine positionality, personal experience, preconceived judgments, and interpretations throughout the study.

We received ethical approval from the Kenya Medical Research Institute (ref: KEMRI/RD/22), the Kenyan National Ethics (NASCOSTI) (ref: 377134), and the Swedish Ethical Review Authority (2024–07452-01–689673). All the data are stored at the Technical University of Kenya per data management regulations, and pseudonymized data are stored on a secure platform at Karolinska Institutet. The Big Q Qualitative Reporting Guidelines (BQQRG) checklist is provided in Supplementary File 4.

## Results

The analysis process generated three main themes and eight sub-themes, as shown in Table 2.

**Table 2:** Themes and sub-themes generated from the analysis

	Theme 1	Theme 2	Theme 3
Themes	Young adolescents’ food choices are shaped by convenience and familiarity, yet the adolescents express an interest in items beyond the familiar	Young adolescents are mostly bound by adult food provision, yet are beginning to have some opportunities to participate independently	Young adolescents value the social dimensions of food, yet they navigate different forms of belonging across family and peer contexts
**Sub-Themes**	Frequent trusted food outlets that are nearby and familiar	Food provision is formed by adult led routines	Emphasize feelings of belonging with both families and peers around food
	Cost constraints are a reality within the food acquisition process	Given some responsibility within family food provision	Navigate food preferences according to the social occasion
	Imagining new food preferences beyond their normal environment	Appreciate exploring food choice during limited autonomous moments	

### Theme 1: Young adolescents' food choices are shaped by convenience and familiarity, yet the adolescents express an interest in items beyond the familiar

The adolescents’ food choices reflect a tension between reliance on nearby, affordable, familiar vendors and a growing curiosity for food beyond their everyday environment.

#### Frequent trusted food outlets that are nearby and familiar

**Fig. 3:**
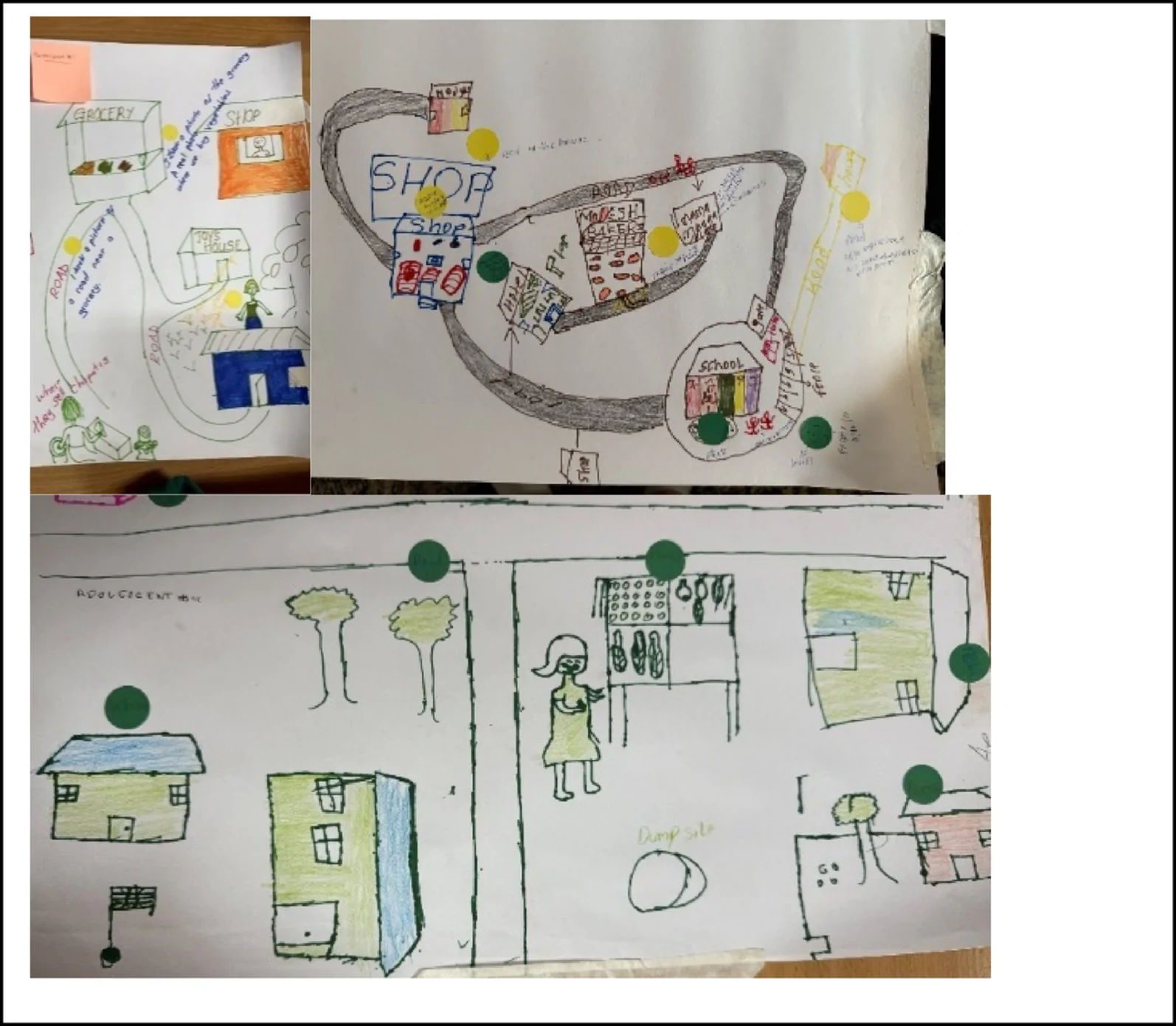
Maps drawn by adolescents showing their food environment as nearby (Top: Group C, P3, girl; Group A, P5, girl; Bottom: Group B, P4, boy)

The adolescents identified food acquisition as occurring in nearby, trusted spaces. They consistently referred to a few familiar locations near their homes and schools. These places were valued for their convenience, cleanliness, and familiarity, fostering a sense of comfort and security within their uncertain environment. Their mapping highlighted young adolescents’ reliance on these trusted food sources within a walkable cluster (Fig. 3). Frequent visits to these few places reinforced adolescents’ trust in and familiarity with them. One girl described,

*“Outside our school…near by the road the mama mboga [food vendor] sells here. When you go along the road*,* you find the shop…down this road*,* you’ll find another shop*,* head straight you’ll return to the school.”* (Group C, P1, girl).

#### Cost constraints are a reality within their food acquisition process

**Fig. 4:**
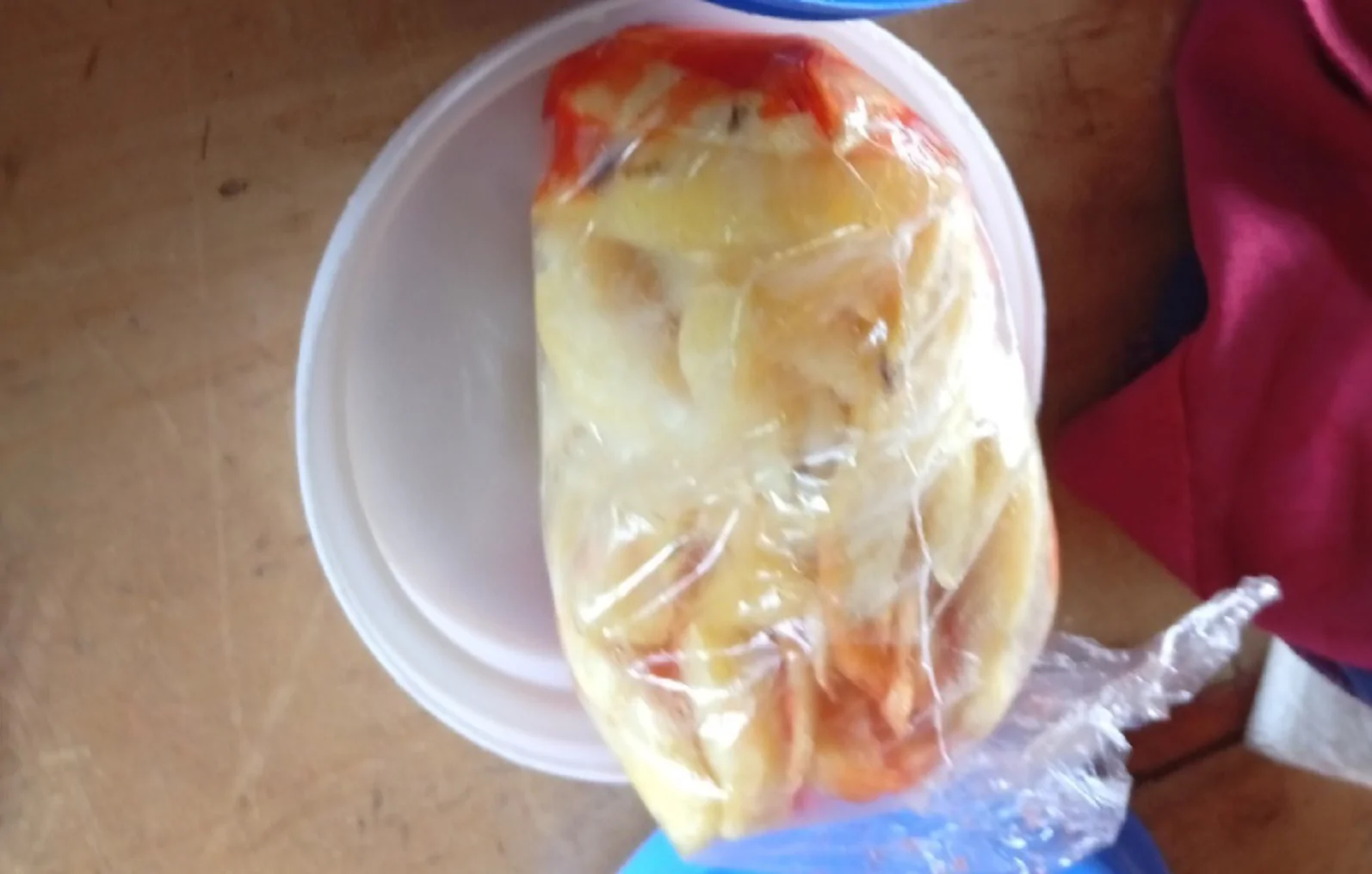
Roadside chips (fries) in a bag with ketchup (Group D, P3, boy)

Cost and convenience were influential factors constraining the adolescents from selecting their preferred foods or eating healthy meals. The adolescents described skipping items such as milk or choosing cheaper options such as roadside chips (fries) due to affordability (Fig. 4). One boy explicitly explained, *“sometimes to eat healthy [foods]*,* it depends [on] the amount of money they get to buy the food”* (Group B, P5, boy). On Saturdays for lunch, adolescents could only afford low-cost items, with a small amount of money provided by their guardians. As one boy explained, he bought rice and beans because *“[the meat options] are very expensive”* (Group D, P3, Boy). This illustrates how cost often outweighs other factors in food choice.

#### Imagining new food preferences beyond their normal environment

Although the adolescents are mostly limited by nearby, low-cost food sources, they aspire to try foods beyond their usual environment, viewing distant vendors or more uncommon items as special. One girl expressed wanting kebabs but said, *“It’s hard to get them near home; we get them far away from home”* (Group A, P5, girl). A boy also described that they only visit the larger, expensive supermarkets for special occasions, placing them outside their routine foodscapes and holding an extra allure.

When asked to imagine independent food choices, adolescents mentioned rarer foods such as “*strawberries*,” “*kebab*,” “*hotdog*,” “*bread*,* jam*,* and boiled egg*,” “*pizza*,*” and “burger*” (Group C, Group D, boys and girls). These items were seen as desirable yet uncommon, making them appealing for their novelty and limited availability. A boy expressed a desire for pizza and noted, *“It is so sweet*,* I have just tasted it ten times”* (Group D, P1, boy).

### Theme 2: Young adolescents are mostly bound by adult food provision, yet are beginning to have some opportunities to participate independently

While adult routines dominate young adolescents’ food provision, small opportunities for independent choice and participation are beginning to emerge.

#### Food provision is formed by adult-led routines

**Fig. 5:**
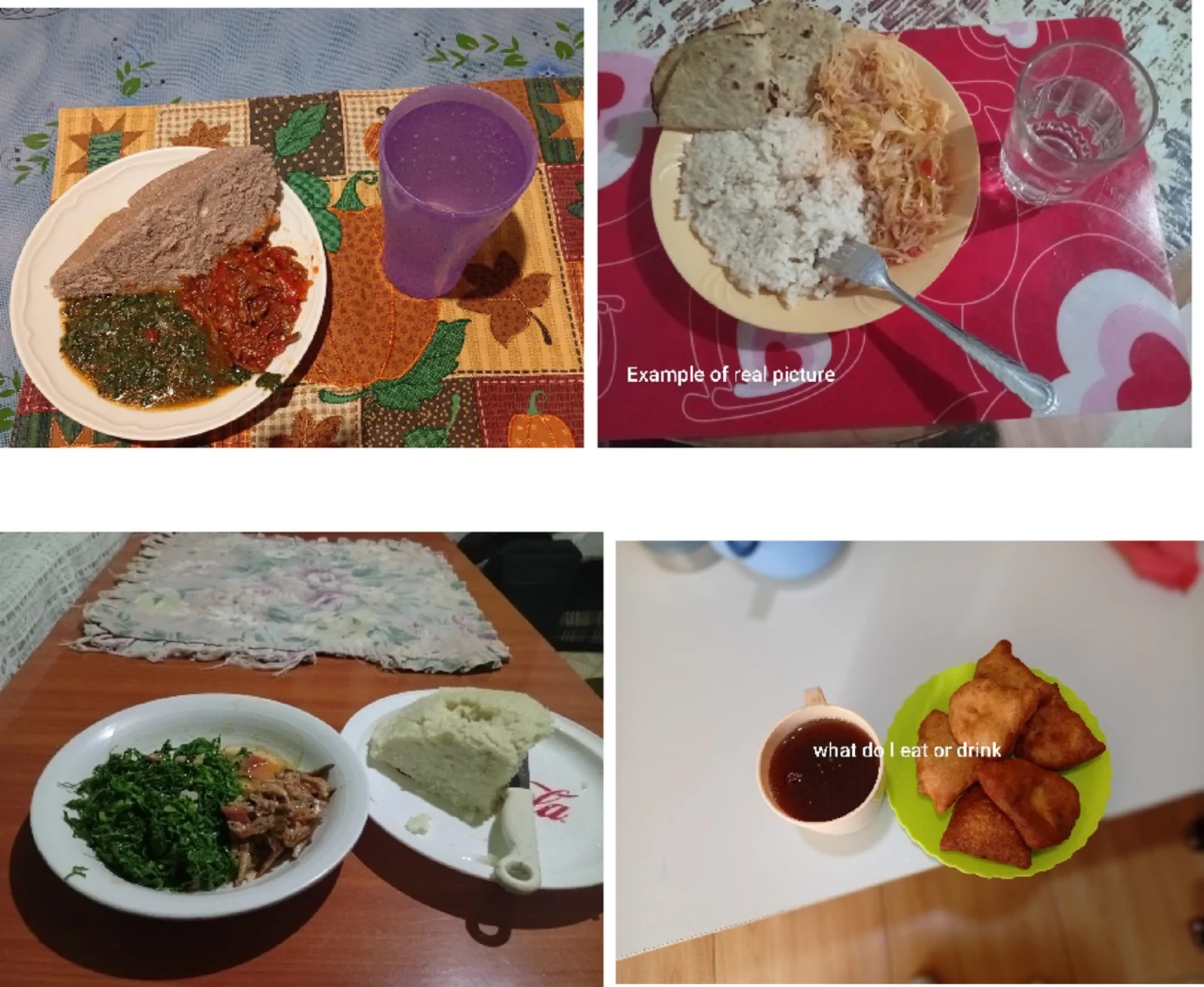
Examples of meals provided at home including ugali (stiff maize porridge), sukuma wiki (leafy greens), omena (sun-dried small fish), chapati (unleavened flatbread), cabbage, rice, and mandazi (fried dough) (Top: Group B, P5, boy; Group A, P2, girl; Bottom: Group C, P3, girl; Group D, P1, boy)

Adolescents followed familiar eating routines at home centered on staple starches, often accompanied by vegetables and occasionally fish or meat, which are typically prepared by their mothers or a female relative (Fig. 5). One girl shared, “*my mother goes to get the food and prepares it for us*” (Group A, P4, girl). Mothers or female relatives are often the main decision makers, purchasers, and preparers of meals, with limited input from the adolescents. Even outside the home, at neighbors’ homes, hotels [informal roadside eateries], or churches, adults were the primary food decision makers, and the adolescents rarely expressed resistance to the prevailing adult decision making.

**Fig. 6:**
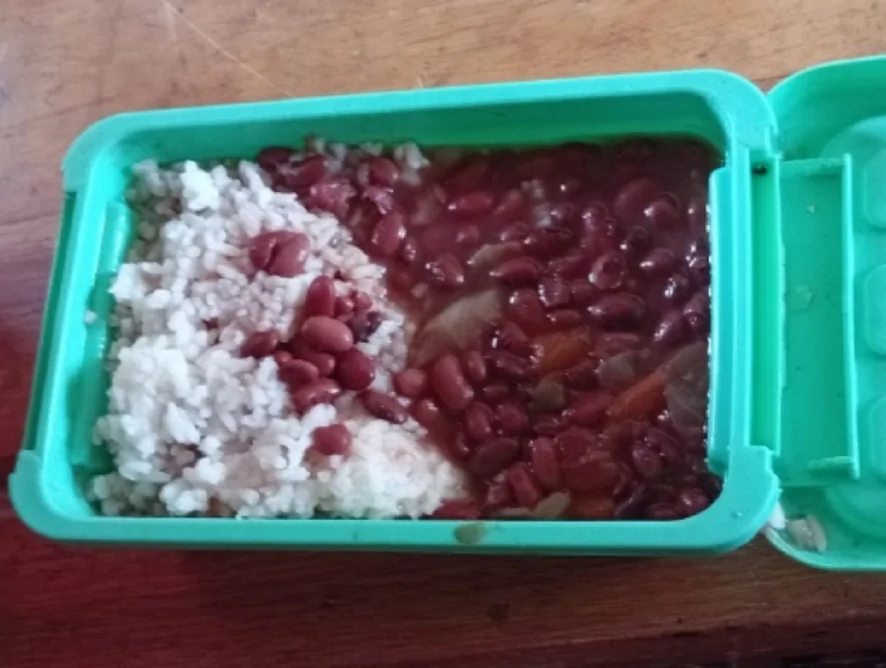
Example of a meal from the school lunch program, including rice and beans in a reusable container (Group A, P1, girl)

Similarly, at school, the adolescents received standardized lunches from a non-governmental program that typically consisted of rice with beans, green grams (mung beans), or bananas (Fig. 6). While the program lacked variety and did not allow for adolescent choice, several adolescents appreciated its reliability and affordability. One boy shared, *“[The lunch program] means so much to me because they have helped us to get a lot of food [for very] little money” (Group D*,* P1*,* boy).* For those who could afford it, the lunch program was essential because without it, they might *“just wait until the time to go home”* (Group D, P4, boy). On the other hand, some did want more variety, but they appeared to hesitate voicing their opinions to teachers, potentially since they did not view school food decision-making as their role.

#### Given some responsibility within family food provision

**Fig. 7:**
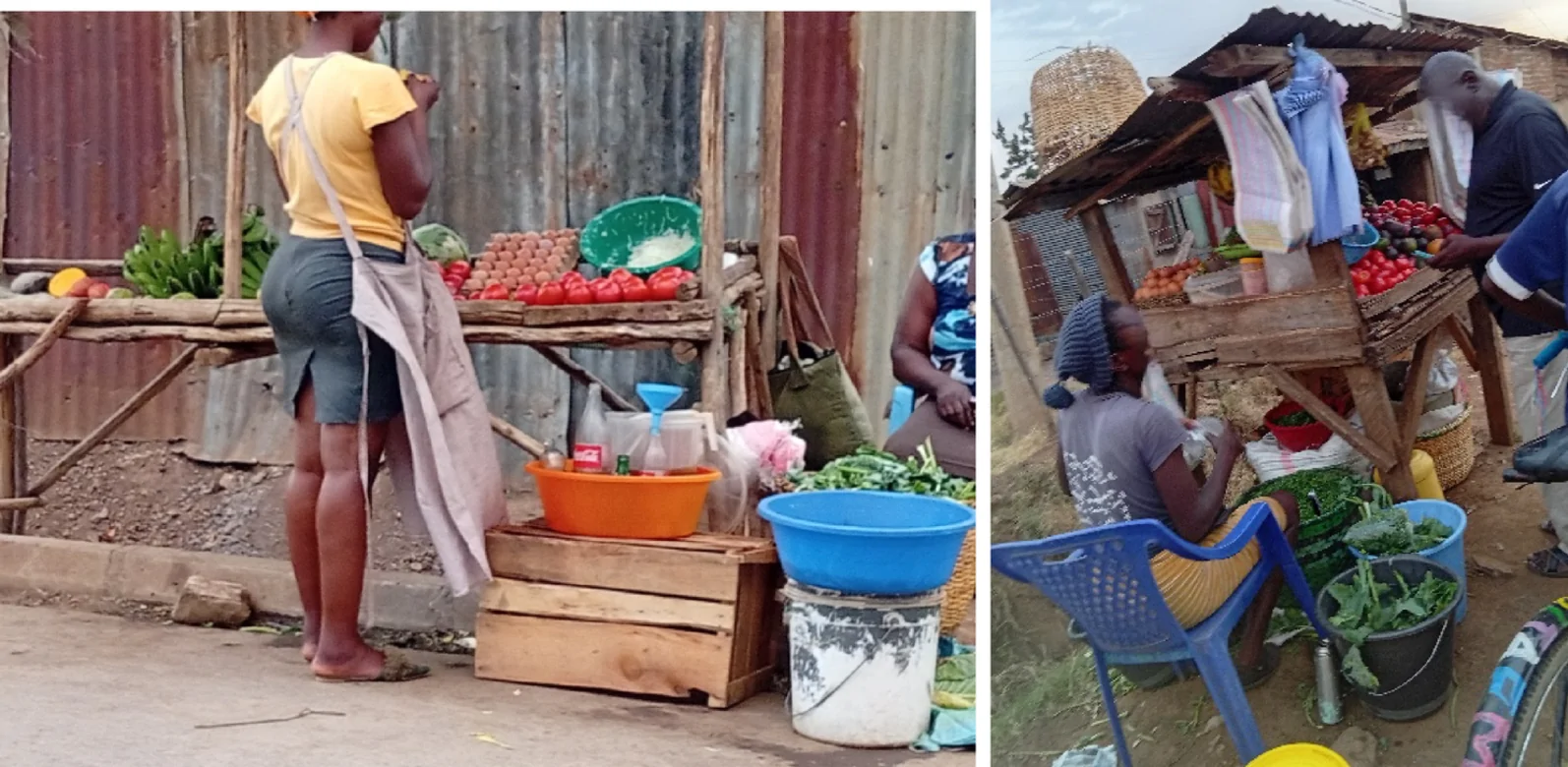
Examples of a mama mboga (food vendor) and a market stall the adolescents are sent to purchase food (Group A, P3, girl; Group D, P2, boy)

Although their influence over food choice was limited, these young adolescents began taking small roles in household food tasks, such as buying from vendors or helping with meal preparation. Several adolescents showed food vendors and described being sent to obtain “*fruits and vegetables*,*”* or “*eggs*,* meat*,* or even indigenous vegetables*” (Fig. 7). One boy discussed, “*there is the mama mboga [food vendor] place*,* where I am normally sent by my grandmother to buy some fruits or vegetables”* (Group D, P3, Boy).

**Fig. 8:**
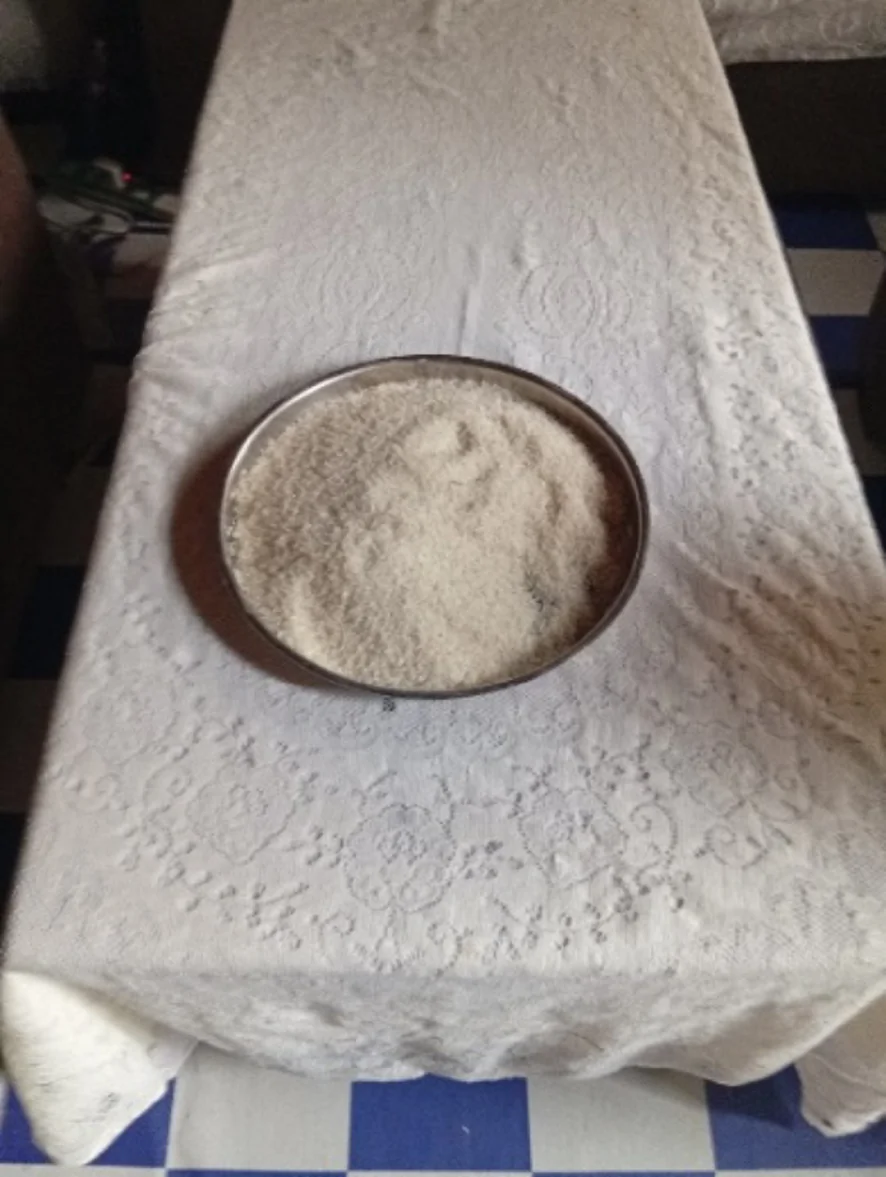
A container of uncooked rice, an adolescent’s grandmother is teaching her how to prepare (Group C, P4, girl)

Girls increasingly took on learning to cook, whereas boys rarely mentioned similar tasks, indicating a gendered pattern of food preparation. During member reflection, the girls joked about the boys struggling with tasks such as cutting vegetables. The boys described participating in other ways, such as running errands or lighting the stove. The girls expressed pride in their cooking skills; one girl showed a bowl of uncooked rice (Fig. 8) and explained how her grandmother taught her to prepare it. Another explained that she can *“cook omena [sun-dried small fish]*,* but ugali [stiff maize porridge]*,* I cannot cook”* (Group C, P2, girl). Through these examples, the adolescents highlight a gradual transition toward greater involvement in food preparation, especially among girls.

#### Appreciate exploring food choice during small autonomous moments

**Fig. 9:**
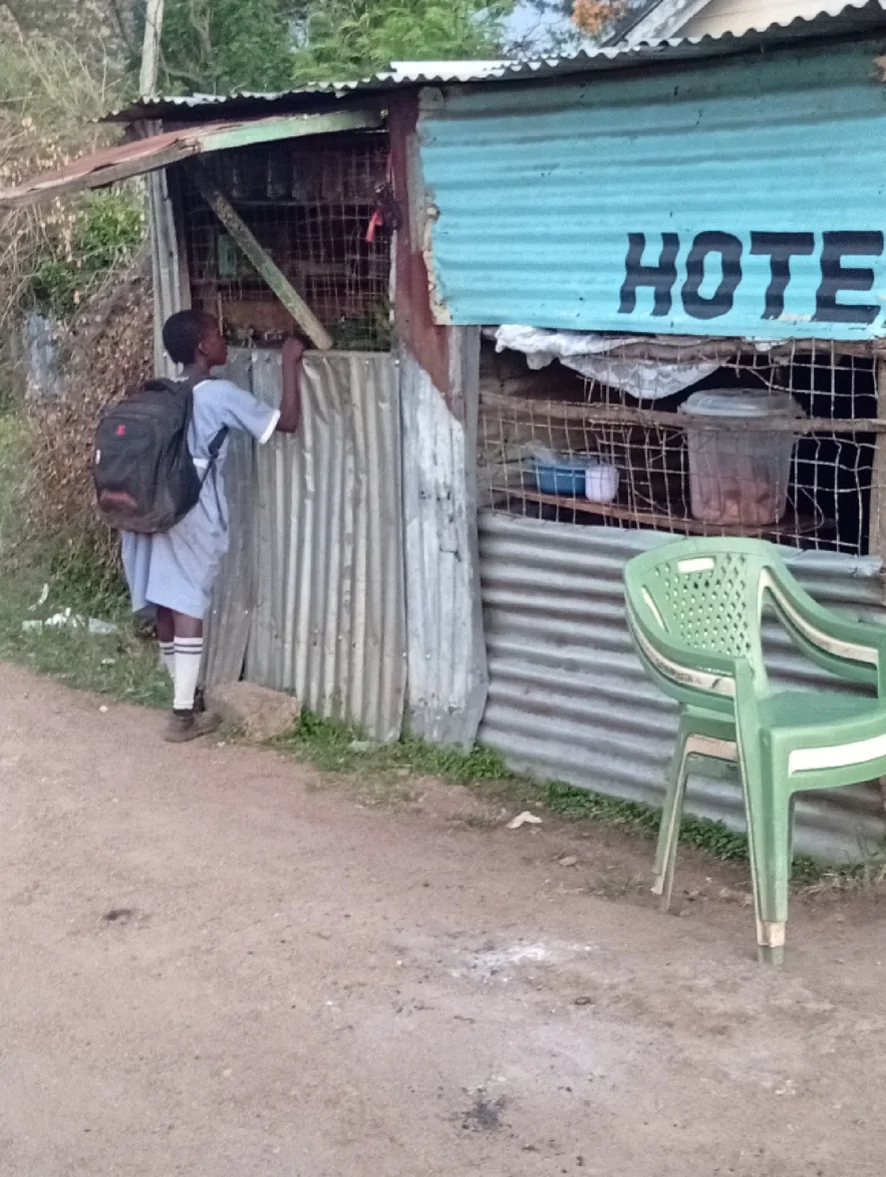
Hotel (informal roadside eatery) where they can go get lunch on Saturdays (Group C, P3, girl)

**Fig. 10:**
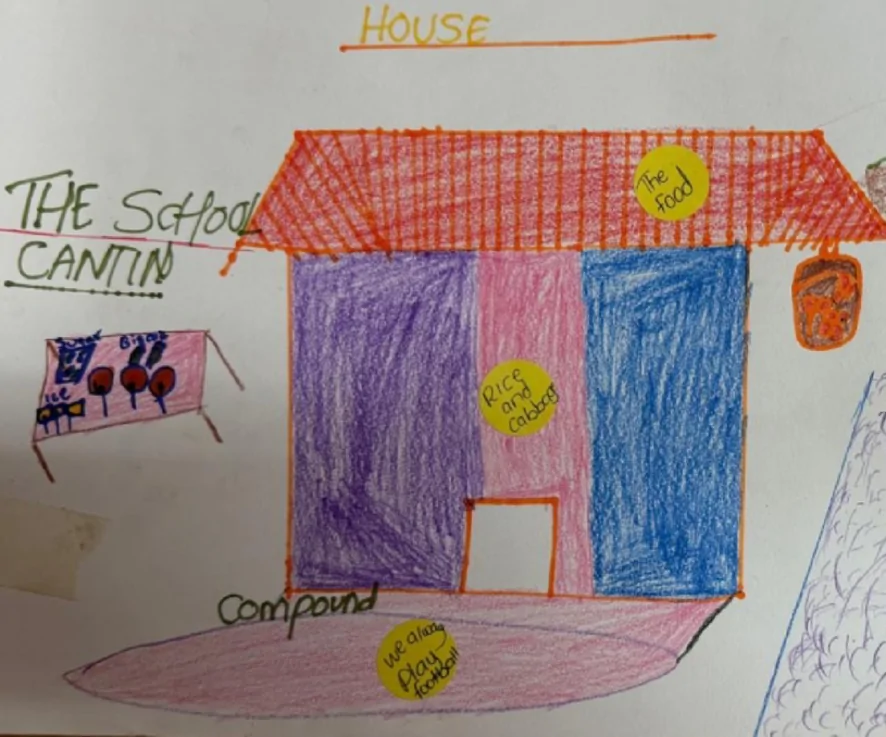
Drawing of an adolescent girl’s house and then the school canteen next to it (Group A, P4, girl)

The adolescents were also granted, by adults, small moments to make autonomous social and food choices. These events occurred mainly during Saturday lunches, short school breaks, or brief stops at local shops on their way home. “*Only on Saturdays*,” it was common for them to be given a small amount of money to buy lunch from small, nearby *“hotels*” (informal roadside eateries) or *“shops and maybe in bakeries*” (Fig. 9). Adolescents also used small allowances to buy snacks such as biscuits, sweets, and sugarcane from the school canteen during short breaks. One girl mapped the canteen alongside her home and no other food vendors, underscoring its importance in her food environment (Fig. 10).

Although a small part of their diet, these purchases appealed due to their novelty and the rare chance to choose freely, offering brief moments of autonomy beyond the purview of the adult decisions. Emphasizing his independent choice for Saturday lunch, one boy emphasized, “*I [go] to the hotel [informal roadside eatery]*,* where I normally buy lunch*,* or anything that I want to buy”* (Group D, P3, boy), and a girl described the rare freedom saying, “*It is the time that we get free to walk around*,* outside the school”* (Group C, P3, girls).

### Theme 3: Young adolescents value the social dimensions of food, yet they navigate different forms of belonging across family and peer contexts

Adolescents negotiate food choices around family norms and peer connections, shaping decisions in varied social contexts.

#### Emphasize feelings of belonging with both families and peers around food

**Fig. 11:**
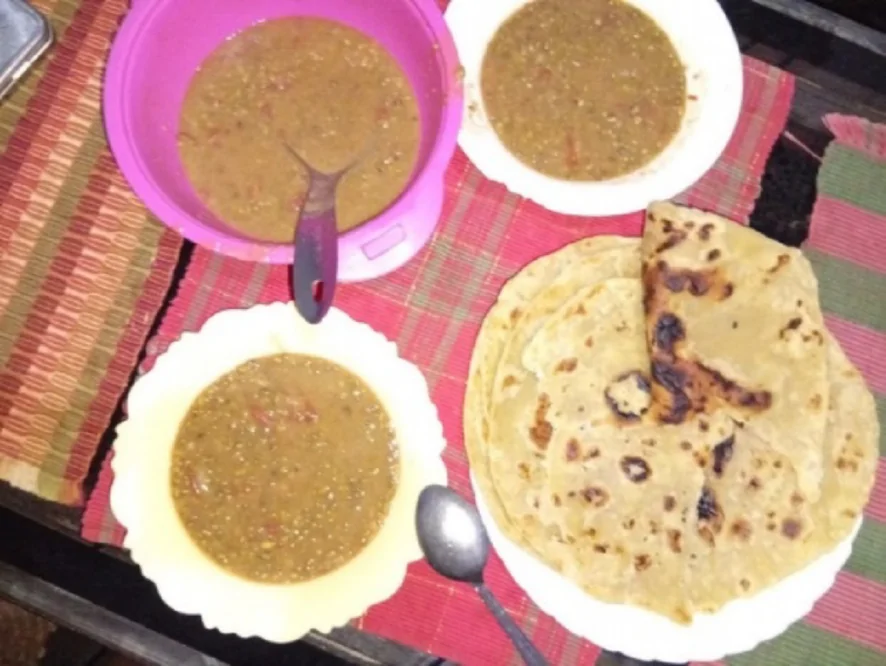
Shared meal of green grams (mung bean) and chapati (unleavened flatbread) (Group A, P5, girl)

**Fig. 12:**
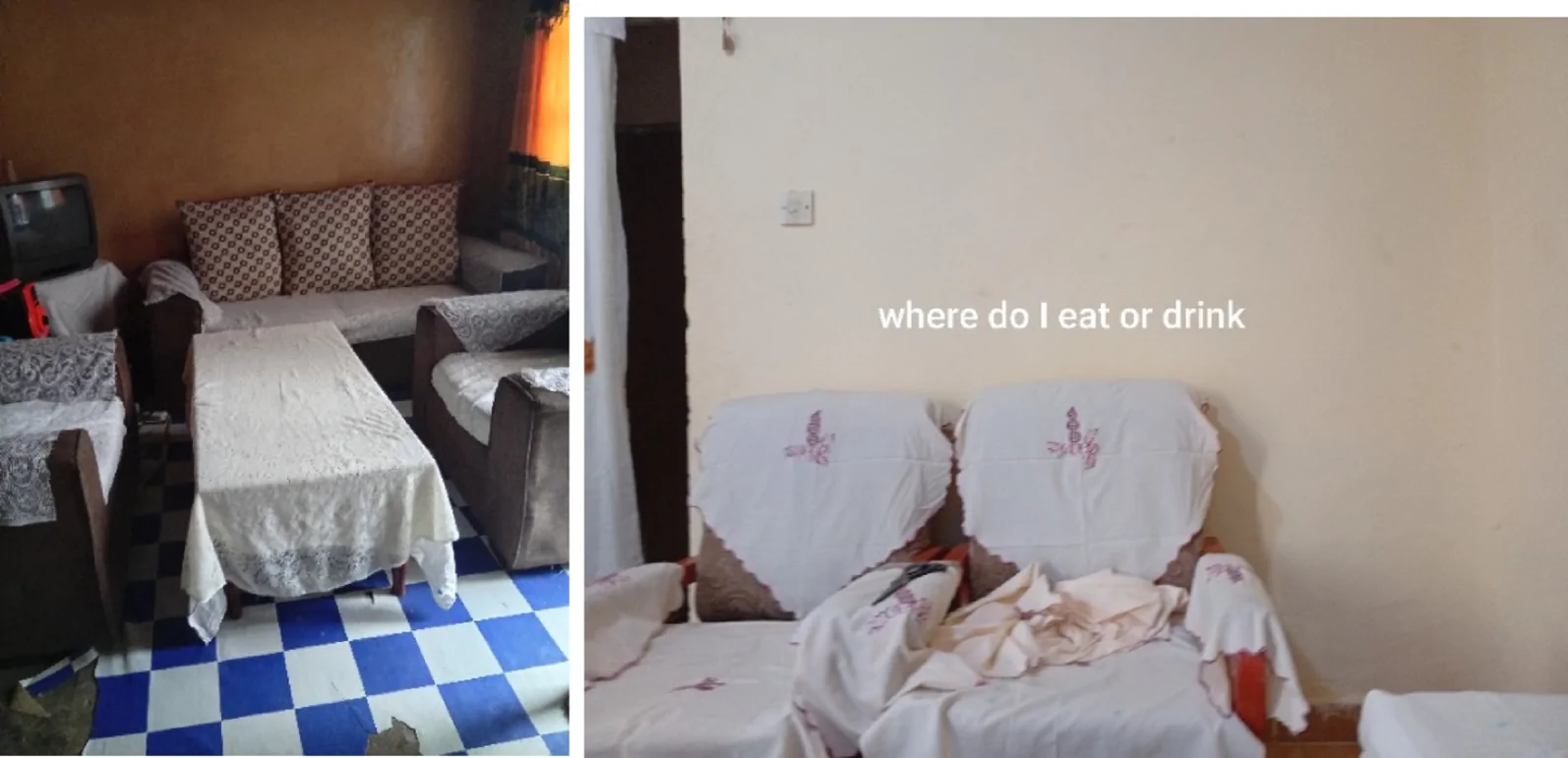
Photos of where they eat with their families (Group C, P4, girl; Group D, P1, boy)

Dinner was described as a time and place for family cohesion. The adolescents appeared to embrace the social aspects of dinner, with many depicting evening meals as moments when relatives gathered to socialize and share stories. Several photos demonstrated communal meals similar to the one shown in Fig. 11. One described that she felt *“excited”* at dinner “*because it is good to have a happy family”* (Group C, P3). Figure 12 illustrates this idea through photos of spaces where families gather to eat. Describing his photo, a boy said, eating with my family *“means a lot to me because we stay together and eat together and make stories”* (Group D, P1).

**Fig. 13:**
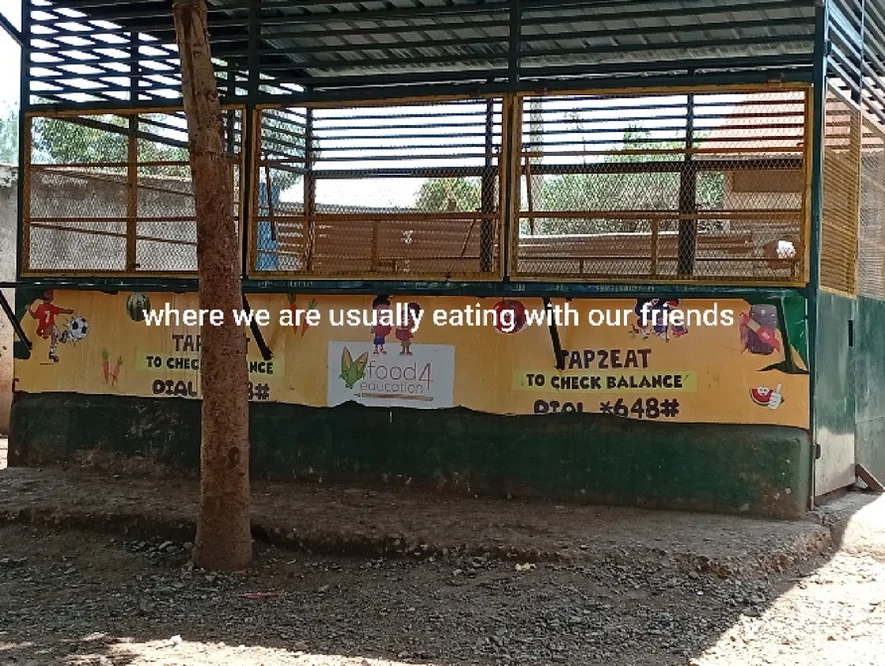
Lunch program stall photo digitally edited by the participant to say, “where we are usually eating with our friends” (Group D, P1, Boy, 12)

Food also served as a platform for socialization with peers. Adolescents consistently described meals and snacks as shared moments for “*play[ing]”*,* “read[ing] together”*,* and “storytelling”.* The lunch stall (Fig. 13) was valued not only for food access but also as a social space. One boy described it as *“the place that I always sit with my friends”* (Group D, P2, boy). Food errands also doubled as social opportunities. During the mapping activity, many of the young adolescents included not only food outlets but also homes, friends, and social interactions in the same narrative. One girl shared, *“This is the road that I walk so that I can go and buy ice cream*,* and this is the grocery…And this is my friend’s house”* (Group A, P3, girl). For these adolescents, the physical spaces of the school lunch stall, shops, or the home dining areas can carry different social meaning impacting their food availability and choices.

#### Adolescents navigate food preferences according to the social occasion

**Fig. 14:**
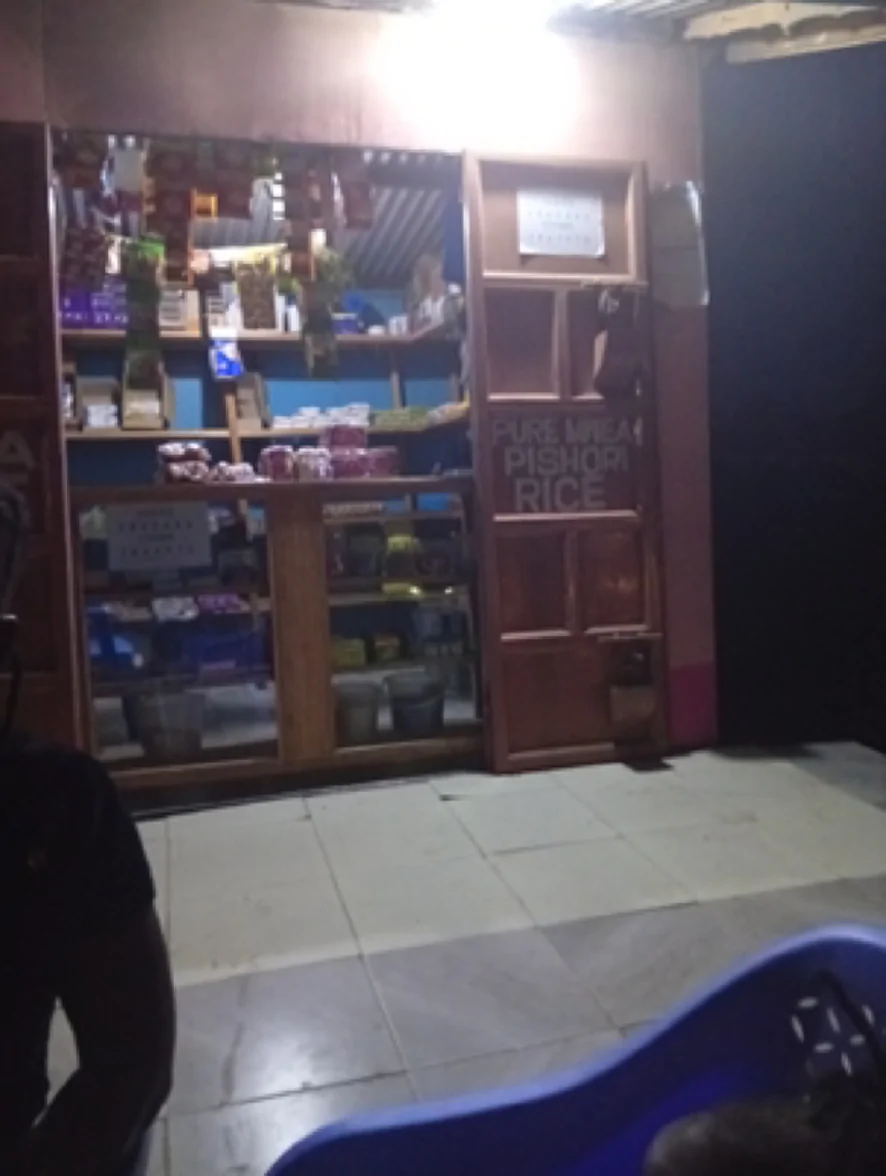
Shop the girl visits with both her mother and friends, but would buy different items depending on who she is with (Group C, P3, girl)

Adolescents tailored food choice to different social contexts, emphasizing more traditional meals with family and more sweets and prepackaged snacks with peers. If making dinner for family, they suggest similar items to what they currently receive at home, but when alone or with peers, they mentioned wanting chips, juice, sweets, lollipops, or biscuits. Encapsulating this contrast, a girl said with friends she *“would buy something like corn puffs*,*”* but with her mom, she would buy *“bread and milk”* (Group C, P3, girl) (Fig. 14). In the member reflection, they elaborated that they would buy healthier foods when with their parents, even if they used their own money, compared with their peers.

**Fig. 15:**
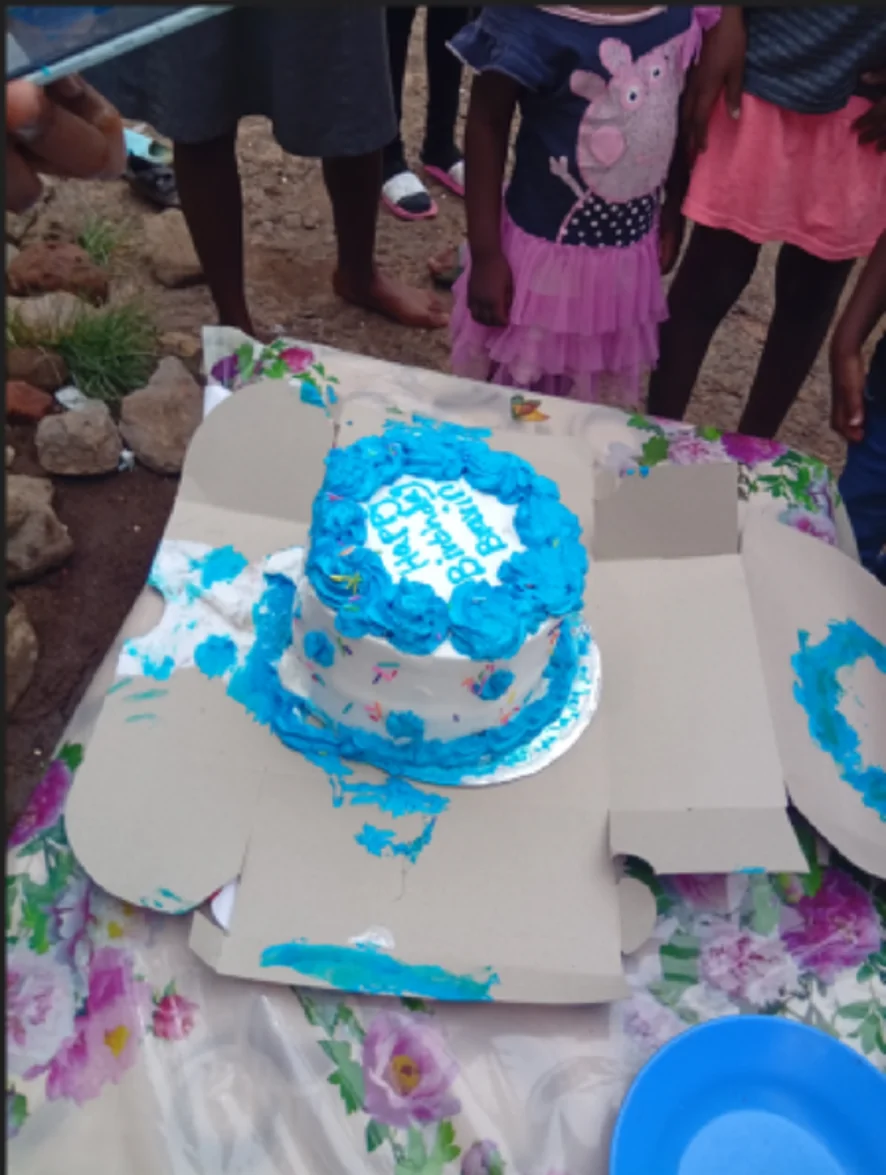
Photo of a birthday cake with many people standing around it (Group C, P2, girl)

The adolescents also associated certain foods with special occasions that carried a strong emotional attachment. Describing celebrations, they emphasized fun and rarer items such as special meats, cakes, biscuits, sweets, soda, and juice. These foods were usually allowed by adults during celebrations, making them exciting and special. Exemplifying this, one boy described, *“this is where I normally go and buy some things during special occasions”* (Group D, P3, boy). Another girl expressed excitement, showing Fig. 15, and described the cake as *“very beautiful and very enjoyable”* (Group C, P2, girl).

## Discussion

Young adolescence represents a pivotal stage for shaping lifelong dietary patterns, and our findings highlight how emerging volitional autonomy interacts with structural and social factors. Drawing on Sobal’s framework (Fig. 1), we illuminate how younger adolescents navigate both macro- and micro-contexts within an urban Kenyan food environment [8]. Examining these dynamics adds nuance to the conceptualization of the early transition period of early adolescence with conditions of persistent adult control and resource-limited settings.

### Macro-context

The young adolescents’ macro-contexts are shaped by spatial and financial constraints. This is similar to other low-income areas, where adolescents perceived their food environment as physically restricted and focused on their immediate surroundings rather than the wider urban or systemic food landscape [52]. Additionally, financial limitations affected both personal and household food choices. This often led to the selection of affordable, potentially less healthy options. This pattern is observed even more explicitly in Nairobi, Côte d’Ivoire, and Zambia, where processed, energy-dense foods are viewed as more convenient, accessible, and more filling for the value [25, 27, 53].

Within these constraints, the adolescents demonstrated a preference for familiarity, trust, and convenience in their food choices. These findings reflect research showing that geographic, cultural, and taste familiarity influence dietary behaviors [52, 54, 55]. Familiarity with food vendors or outlets fosters trust, which can influence purchasing decisions related to both food safety and feelings of social trust [28, 56]. Their reliance on trusted vendors underscores how young adolescents navigate by prioritizing familiarity and safety in the potentially unsafe, unhygienic, and at times uncertain environment.

### Micro-context

The micro-contexts of adult-controlled food provision and the importance of social values also shaped adolescents’ food choices. According to the global literature, younger adolescents’ food provision remains largely shaped by adult-led routines at home and school [57–59]. These routines provide platforms that emphasize social connection and norms through food choice. The social food environment and routines have been shown to influence dietary habits and connect adolescents with people, places, and events [21, 25, 28, 54]. Consistent with other studies, our study showed that shared meals at home foster familial connection [52, 54, 60], while food with peers is a way that adolescents seek belonging and independence [1, 25, 27]. The adolescents expressed gratitude for these social dimensions around food and reliable food provision from adult-led routines.

Consequently, the adolescents rarely overtly questioned or showed signs of resistance to adult routines. This contrasts with other evidence from Africa, the US, and Europe, where adolescents have been observed to challenge adult-managed food routines and instead favor peer norms, often involving the purchase of energy-dense foods [4, 22, 25, 27, 59]. Although we are unable to know for sure, we postulate that this could be explained by their heightened appreciation of food accessibility within their lower-socioeconomic context, their awareness of food insecurity, the local culture of obedience for adolescents, their younger developmental stage, or a mixture of all these considerations [6, 41]. These findings resonate with a U.S.-based model that depicts a core circle of adult routine and control with a smaller transitional zone of emerging autonomy around it [59].

### Adolescent transitional phase and autonomy

The young adolescents began to embrace some autonomy within acceptable adult boundaries by imagining alternative food choices and appreciating moments of independence. These small independent choices often reflect both peer-influenced food choices and food prestige. Other studies suggest that foods seen as non-traditional, energy-dense, or Western-associated often carry prestige and signal social status [6, 61, 62]. Specifically, a photovoice study in which older adolescents in Nairobi preferred ultra-processed, high-fat foods because they are considered cheap, convenient, youthful, and modern, while home-provided meals were considered boring, rural, and associated with older generations [27]. Food prestige appears in our findings alongside appreciation of traditional foods. Yet, evidence from other contexts suggests that as adolescents grow and gain autonomy, their choices may increasingly mirror patterns where modern, energy-dense foods signal status and desirability, and overshadowing traditional foods.

Although adolescents’ food choices may diverge from dietary guidelines, they are in a critical developmental stage for practicing independent decision-making and fostering skills essential for adulthood [1]. Our findings suggest that these young adolescents take pride in moments of independent choice, enjoy selecting foods with peers and desiring greater influence over school meals. This aligns with other literature that even in resource-constrained settings such as Bangladesh, Ethiopia, and the Gambia, food serves as a means for adolescents to express their autonomy [1, 4, 21, 53].

According to developmental psychology research, the emergence of autonomy is a key aspect of adolescent wellbeing [63]. Interestingly, the only major difference between the gender-specific groups was the split responsibilities regarding food preparation. Specifically, volitional functioning autonomy, defined as acting in alignment with one’s own values and interests, is essential for wellbeing and intrinsic motivation [63]. Fostering autonomy through food-related decisions can strengthen self-endorsed motivation and support healthier choices [63]. Our findings show that these young adolescents are learning to navigate food choice contexts such as proximity, cost, gender roles, social meaning, and desirability, and enhancing volition autonomy can support their personal decision making.

### Implications for practice and research

The young adolescent-centered approach of this study served to advance the conceptual understanding of early adolescence as a key transition moment when action can be taken to promote healthy diets across the life course. First, creating enabling food environments for these adolescents could consider not only accessibility and affordability, but also leveraging the familiarity with trusted food sources to enable healthy behavior. Second, among these younger adolescents in Kisumu, guardians and schools remain pivotal influencers and should be closely involved in any efforts to influence the adolescents’ food environments. However, this should not undermine the development of their autonomy and learning to navigate food choice. In that same vein, third, our findings suggest that these adolescents valued moments of autonomous food choice, which may support skills to negotiate contextual influences, foster healthy food choices, and influence life-course dietary patterns [27, 28].

Further research could employ longitudinal studies on adolescents to examine food choices and mixed methods approaches linking perceptions with dietary data. Additional areas of interest include exploring gendered expectations, peer dynamics, and food prestige and co-developing interventions rooted in adolescents’ emerging autonomy [27, 28]. Recognizing adolescents as active partners is an essential part of any future research. They bring lived expertise and creative problem solving that can be more inventive than adult thinking [53].

### Strengths and limitations

There are several strengths to this study, namely, the innovative photo elicitation and social foodscape mapping methodology. This provided interactive, context-rich visual and narrative data and allowed for crystallization across the various data sources. Using rapport-building activities, gender-specific groups, and a multi-lingual facilitation approach enhanced adolescent expression and further strengthened the data collection process. Additionally, the facilitator team included younger local facilitators to support open participation. Lastly, we included both insider and outsider perspectives in the analysis and conducted a member reflection, which supported the trustworthiness of the findings.

However, there were several limitations. The first limitation was potential selection bias from the teacher-led recruitment. The criteria for the recruitment were difficult for teachers to operationalize among many interested adolescents. We were thus unable to confirm the variability in distance from school or the socioeconomic proxy. To address this, we compared these findings with other project data, including adolescent interviews, other photo elicitation data, and workshop outputs, and found no major inconsistencies. Another limitation was the risk of social desirability bias, shaped by the cultural and hierarchical context, which we attempted to mitigate through non-personal questioning, formulating hypothetical scenarios, and the involvement of younger local facilitators, though some bias remained.

## Conclusions

Bringing these insights together, these young adolescents in Kisumu are navigating a critical early transition in food choice, shaped by financial constraints, physical accessibility, and adult-led routines. While adults remain central in shaping their diets, the adolescents’ express pride in moments of independent choice and a desire for greater influence, signaling the early development of volitional autonomy within social and environmental constraints. These findings underscore early adolescence as a critical intervention point to equip the young adolescents with the skills to negotiate their own healthy food choices, thereby influencing lifelong dietary patterns.

## Supplementary Information

Supplementary Material 1

Supplementary Material 2

Supplementary Material 3

Supplementary Material 4

## Data Availability

The datasets generated and analyzed during the current study are not publicly available due to the inability to guarantee privacy and data protection of pseudo anonymized qualitative data but are available from the corresponding author upon reasonable request.

## Abbreviations

AAO: Alice Achieng Ojwang
DN: Donvan Nunda
EG: Elisa Gobbo
EdO: Edward Ouko
EsO: Esther Oruko
HMA: Helle Mølsted Alvesson
JOA: Joseph Ochieng Amoke
KSA: Kristi Sidney Annerstedt
NCD: Non-communicable diseases
SHvW: Sibylle Herzig van Wees
