# Supplementary material for: “It’s where I buy lunch or anything that I want to buy”: a participatory photo elicitation study on adolescents’ experience of food choice and environment in Kisumu, Kenya
Source: J Health Popul Nutr. 2026 Aug 24;45:190. doi: 10.1186/s41043-026-01421-8 (PMC13505041; doi:10.1186/s41043-026-01421-8)
Supplement: Supplementary file 1 [file 41043_2026_1421_MOESM1_ESM.pdf]

Supplementary File 2

Storyboard for Session #2:

Time	Step (What and Why)	Activity (How)	Guiding Questions	Materials
15 mins	Welcome – explain and rapport building	• Present purpose<br>• Voluntary participation<br>• Short Initial discussion and process	• How did it go?<br>• Did the cameras work?<br>• Anything that stood out immediately?<br>• Any challenges?	Consent forms from meeting 1
10 mins	(Optional) Photo selection	• Have the adolescents select the 5 photos that they would most like to discuss	• Please take 10 minutes to select 5 photos<br>• Which ones represent where, how, and what you eat?	Phones from data collection<br><br>Charger
45 mins	Photo Discussion – get a sense of the photos they took, begin understanding of how they perceive their food environment	• Go around sharing photos and discussing the significance	• Please share with us a few of the photos you decided to take and tell us about them<br>• Why did you decide to take that photo?<br>• What does it mean to you?<br>• How are your photos similar or different from your peers?<br>• Do you feel that this was something you chose?<br>• If/when you were in charge of getting food for yourself, where would you go?<br>• How does this (refer to specific photo) impact what you decide to eat?<br>• Were there photos you wanted to take but where unable to?<br>• Are there any differences between the school and non-school days?<br>• Similar or different to a regular day (same as last week?)<br>• SHOWeD framework to guide for probing:<br>o What do you see here?<br>o What is really happening?<br>o How does this relate Our lives?<br>o Why does this condition exist?	Phones from data collection<br><br>Charger

			○ What can we do about it?<br>Hypothetical Ideas<br>• If a friend in the school was given some money (15-20 bob) to go to the market, or a vendor, or Quickmark, what would they go out and buy?<br>• If you (or a friend) could go and get whatever you wanted to eat or drink what would you buy?<br>• If a friend were to go to get something special, what would they go buy?<br>• Imagine that today is a special day and your parent is willing to treat you with fun snack, what would you want to buy?	
30 mins<br>(Note: if the discussion is going really well can ask if they would like to keep discussion and then be interested to meeting a third time for mapping)	Mapping – see how the adolescent spatially understand/ perceive their food environment	• Either individually or collaborative as group (based on preference of adolescents) have them draw a big map of the areas where they took the photos<br>• Mark with a number or description where you would put your photo on the map (utilize stickers as in Logie, et al 2013)<br>• Discuss around mapping	• Are there certain areas that stand out?<br>• Are there spaces you all seem to eat?<br>• How do you feel about the map? Does it feel true to where/how/what you eat?<br>• What does it mean to you?<br>• Are there aspects of the map you would like to change?	Flip chart paper, markers
