# Supplementary material for: “It’s where I buy lunch or anything that I want to buy”: a participatory photo elicitation study on adolescents’ experience of food choice and environment in Kisumu, Kenya
Source: J Health Popul Nutr. 2026 Aug 24;45:190. doi: 10.1186/s41043-026-01421-8 (PMC13505041; doi:10.1186/s41043-026-01421-8)
Supplement: Supplementary file 2 [file 41043_2026_1421_MOESM2_ESM.pdf]

CHANGE MAKER

Photo Study with Adolescents
Member Check

These are our thoughts – Let us know yours!

Take a look at this pictures and tell us what you think

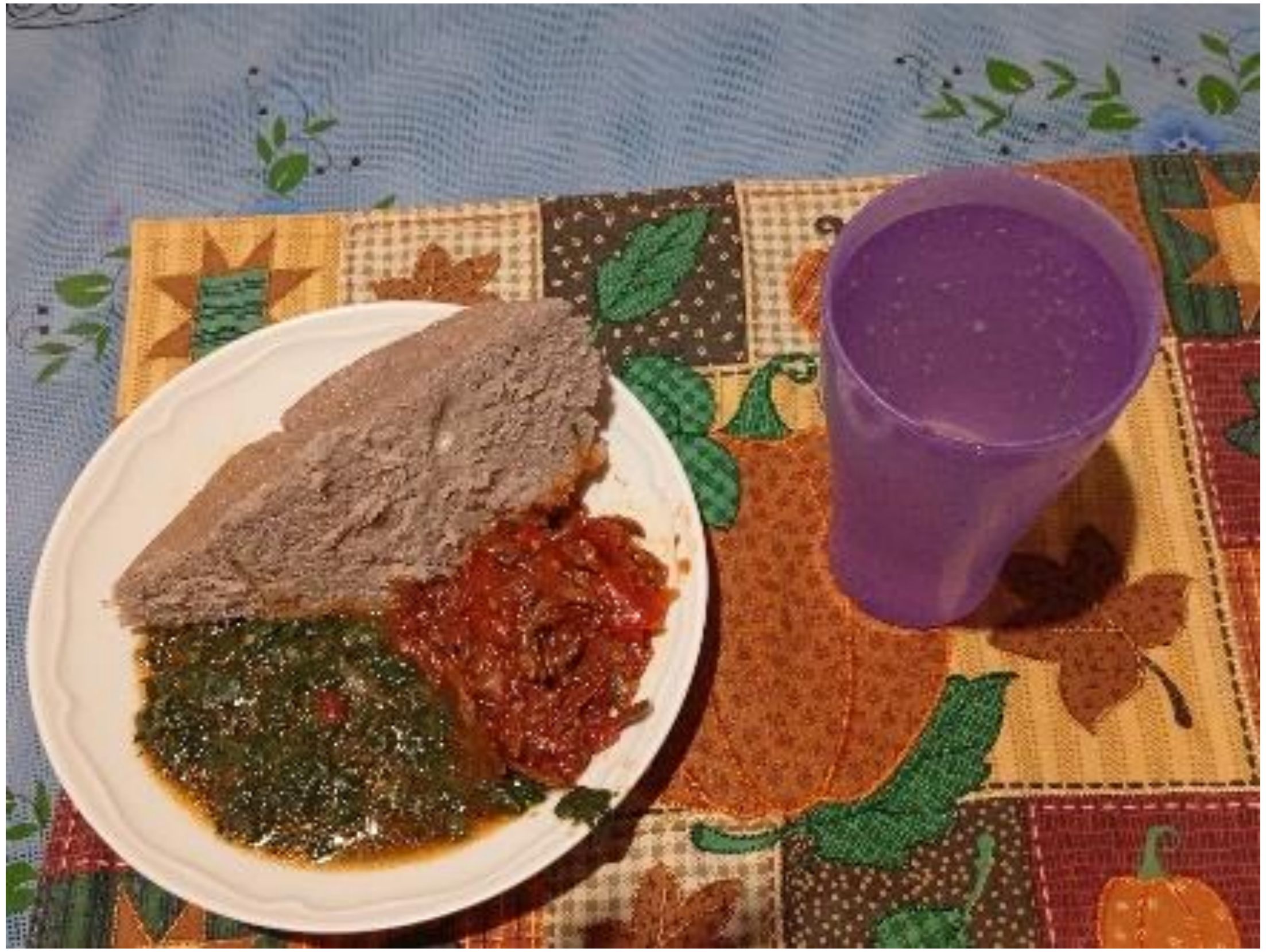

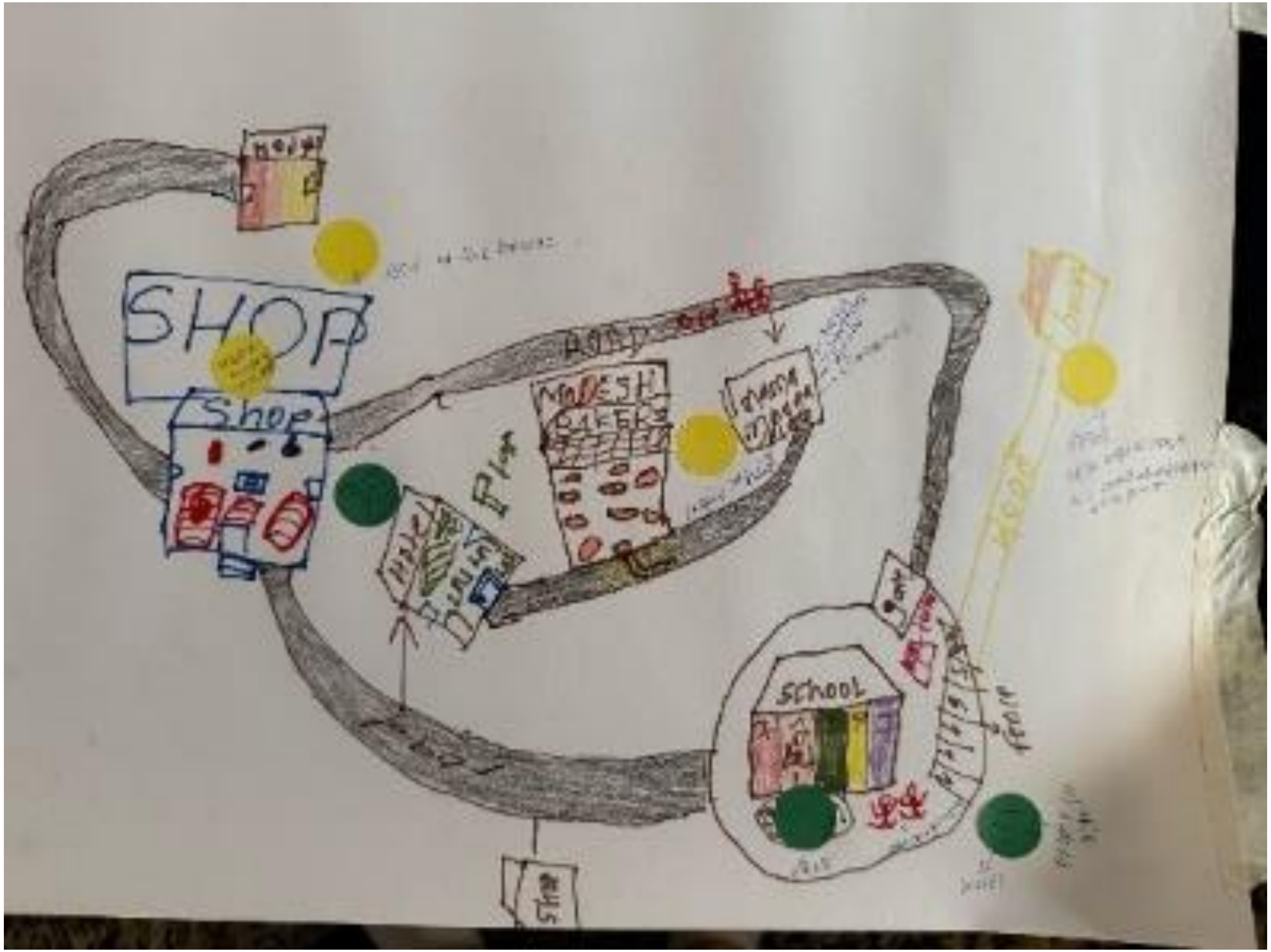
SHOP
Shop
ROAD
Plot
school

Theme 1

- The places that you get food are mostly close to home or school and places you go a lot feel comfortable to you
- Your parents or school give you most of the food you eat.
- Costs effects what you or your families can buy

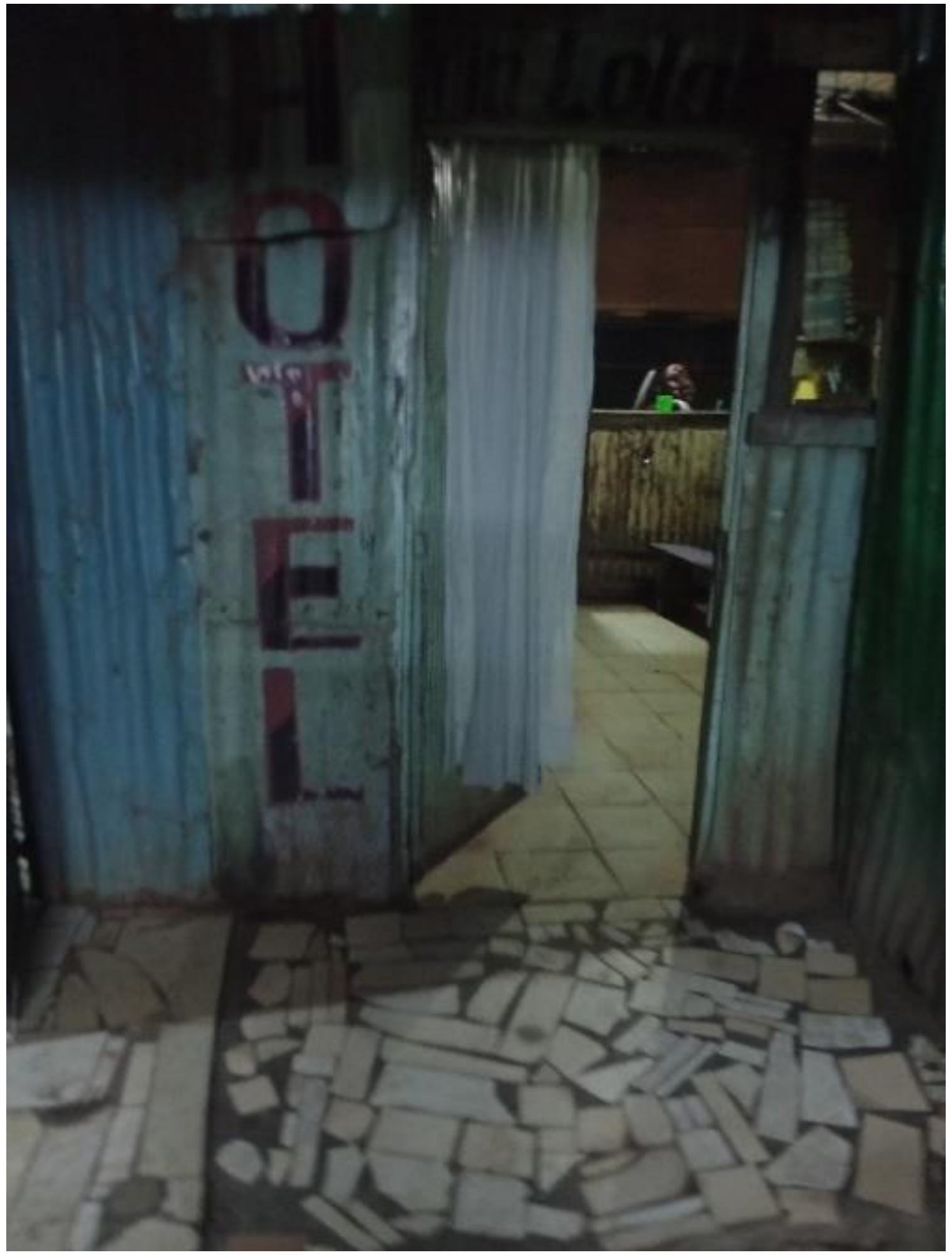

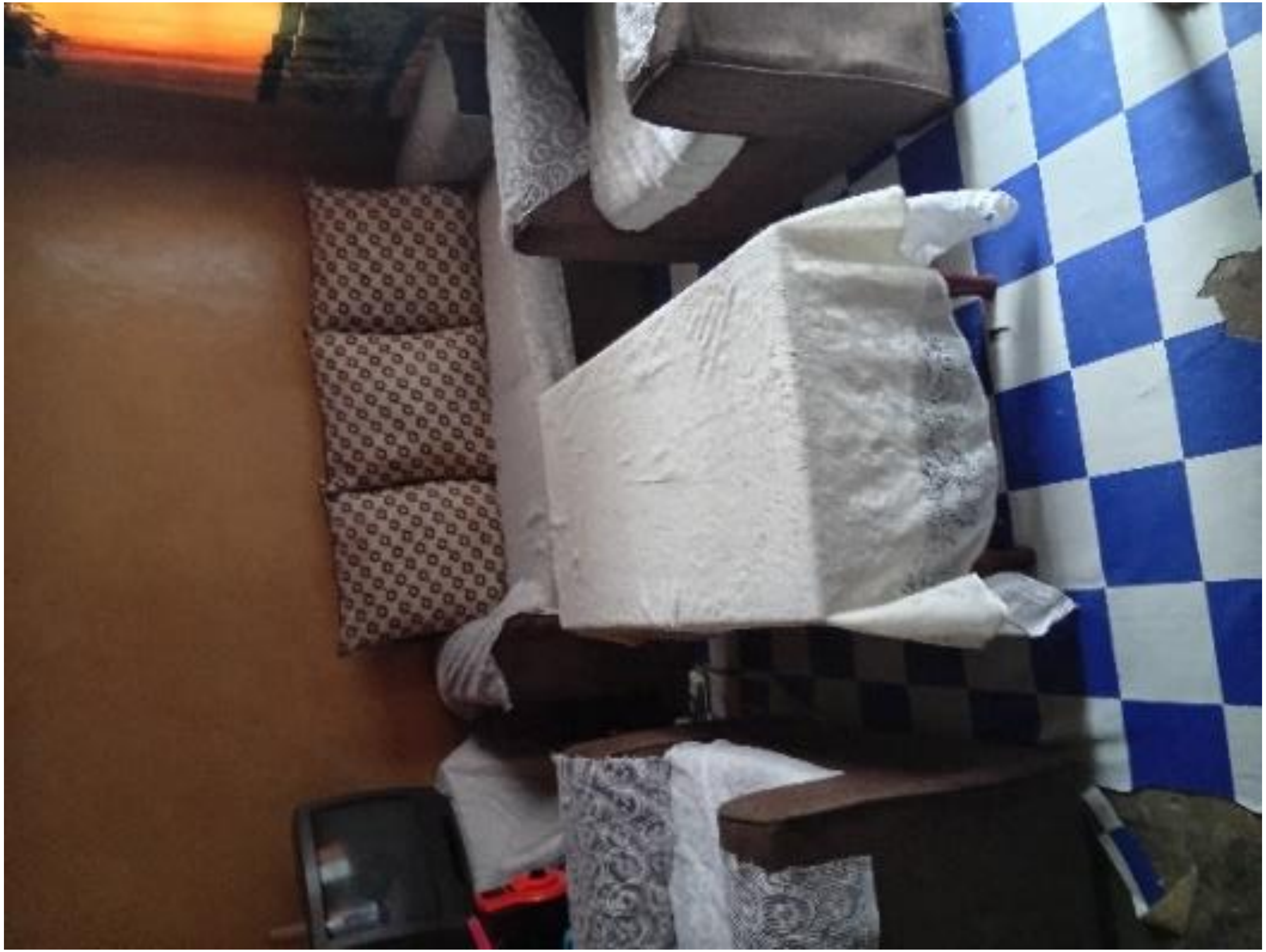

Theme 2

- You have some responsibilities at home to help with getting or preparing food

- You enjoy having some free/independent time to buy lunch on your own on Saturdays or to buy snacks at the canteen or on your way home

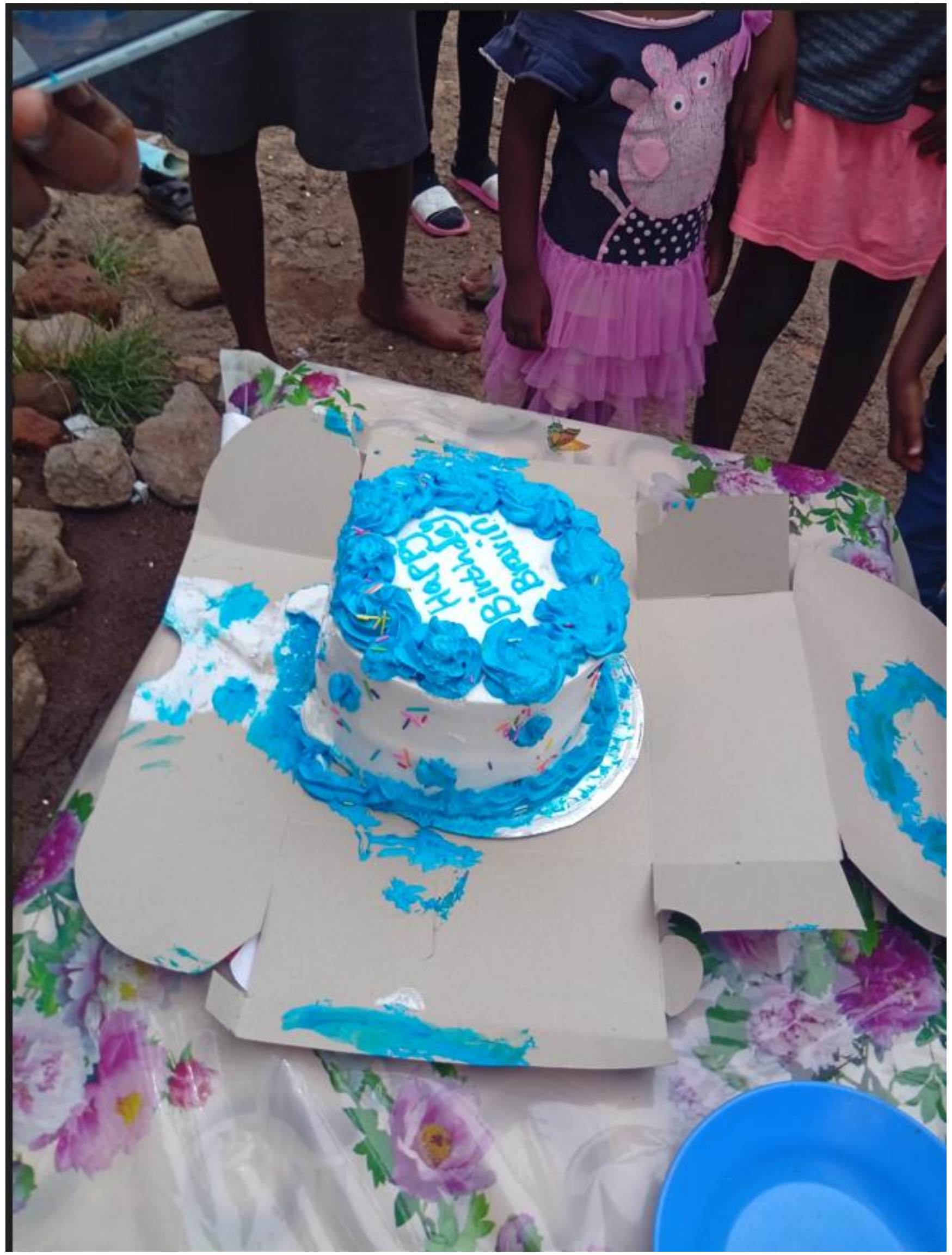

Theme 3

- Hanging out with family and friends is a fun and exciting part of when you eat a meal

- You sometimes eat or buy different things with your friends compared with your mother or care giver

- If you could get anything you want to eat you sometimes want fun items you can't get everyday
