# Supplementary material for: “It’s where I buy lunch or anything that I want to buy”: a participatory photo elicitation study on adolescents’ experience of food choice and environment in Kisumu, Kenya
Source: J Health Popul Nutr. 2026 Aug 24;45:190. doi: 10.1186/s41043-026-01421-8 (PMC13505041; doi:10.1186/s41043-026-01421-8)
Supplement: Supplementary file 3 [file 41043_2026_1421_MOESM3_ESM.pdf]

CHANGE MAKER

Photo Study with Adolescents

Types of Photos

Real – depicts what you see

Symbolic picture – using something to represents what you mean/ feel/ see

Arranged – setting up a photo to have a meaning

Examples of real pictures

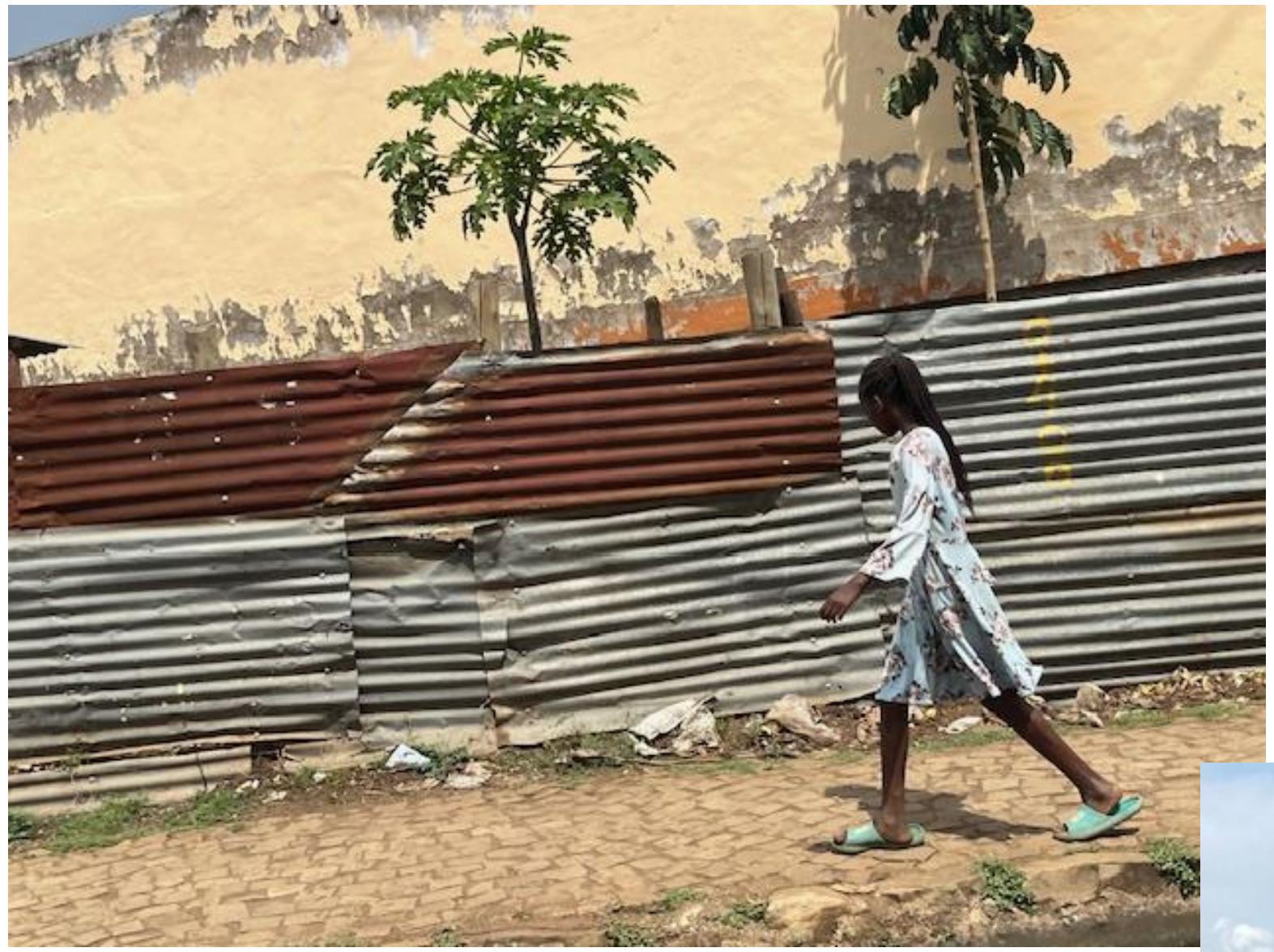

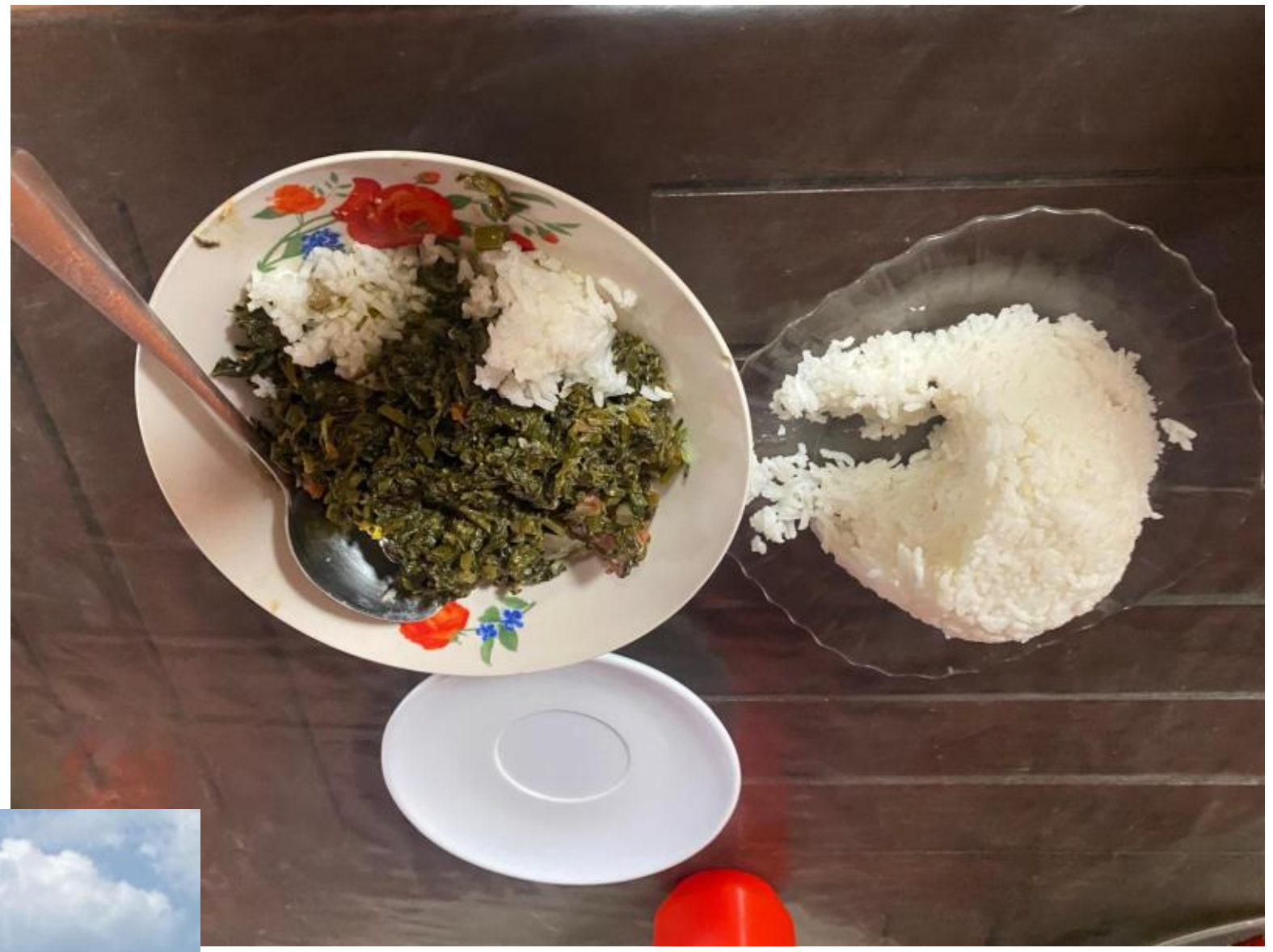

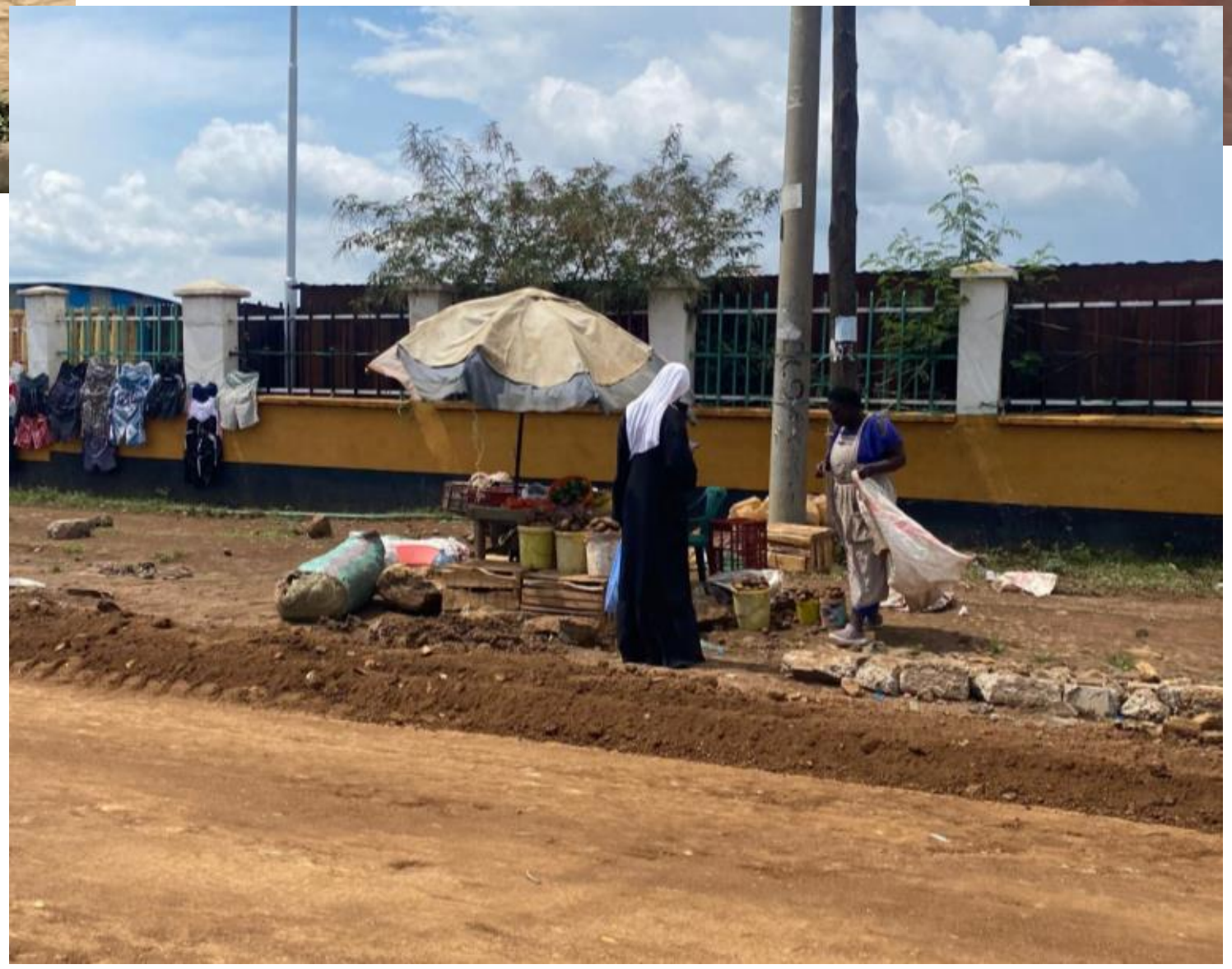

An example of a symbolic picture

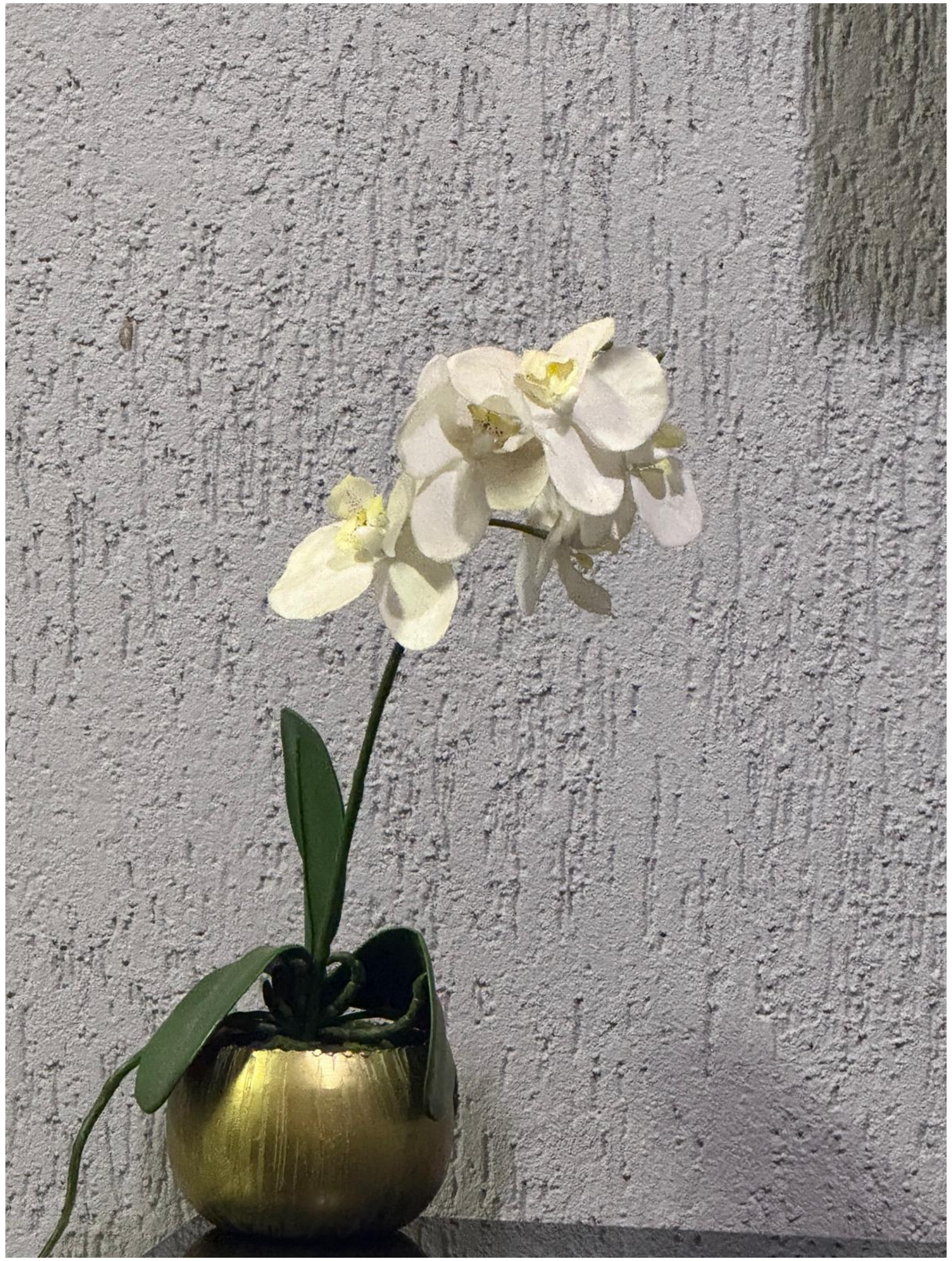

Feeling energy to grow: symbolic illustration

- ***I feel that a lot of the food that I eat is quite fresh and gives me energy to grow like the flower.***

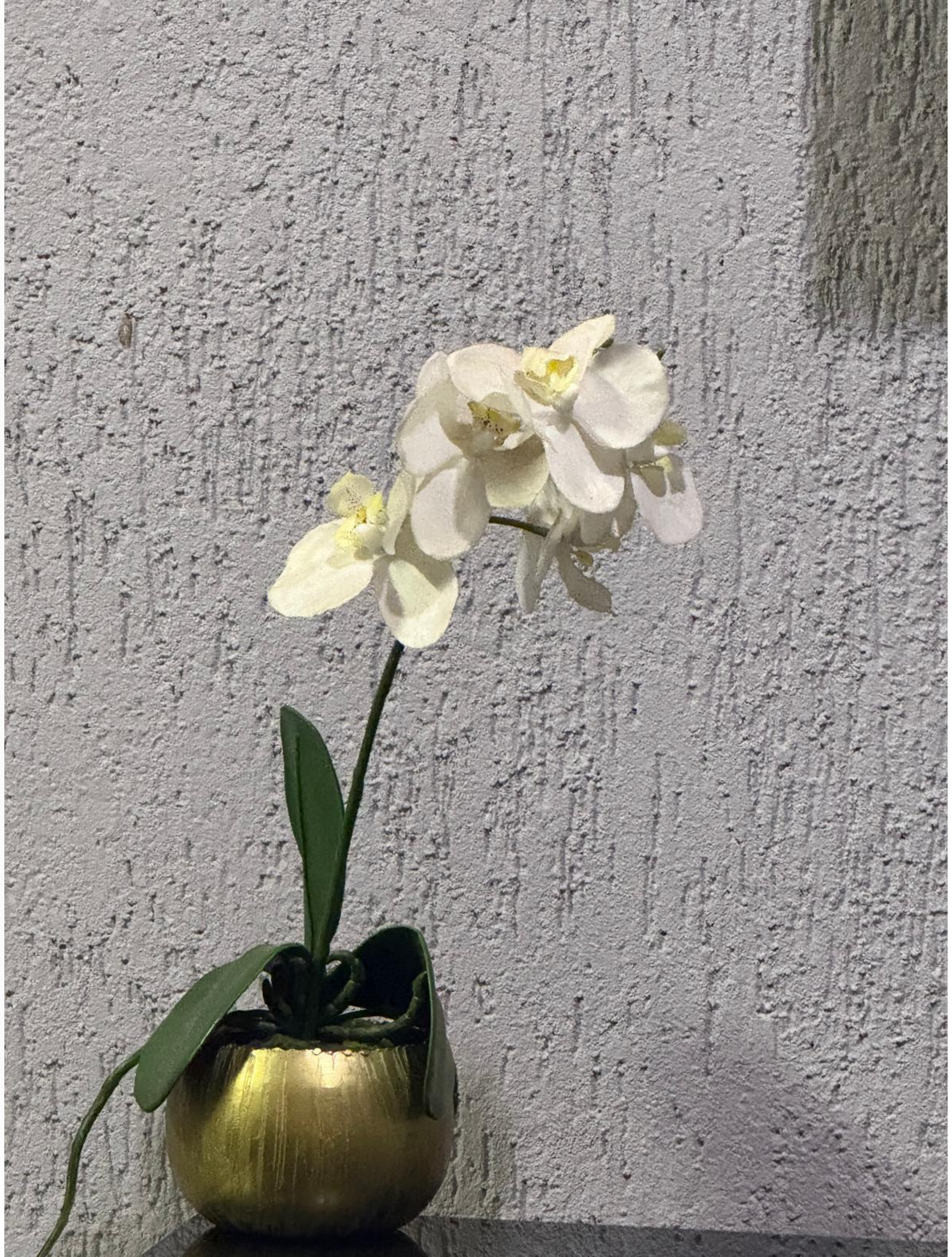

An example of an arranged picture

This pictures shows sitting around the dinner table together.

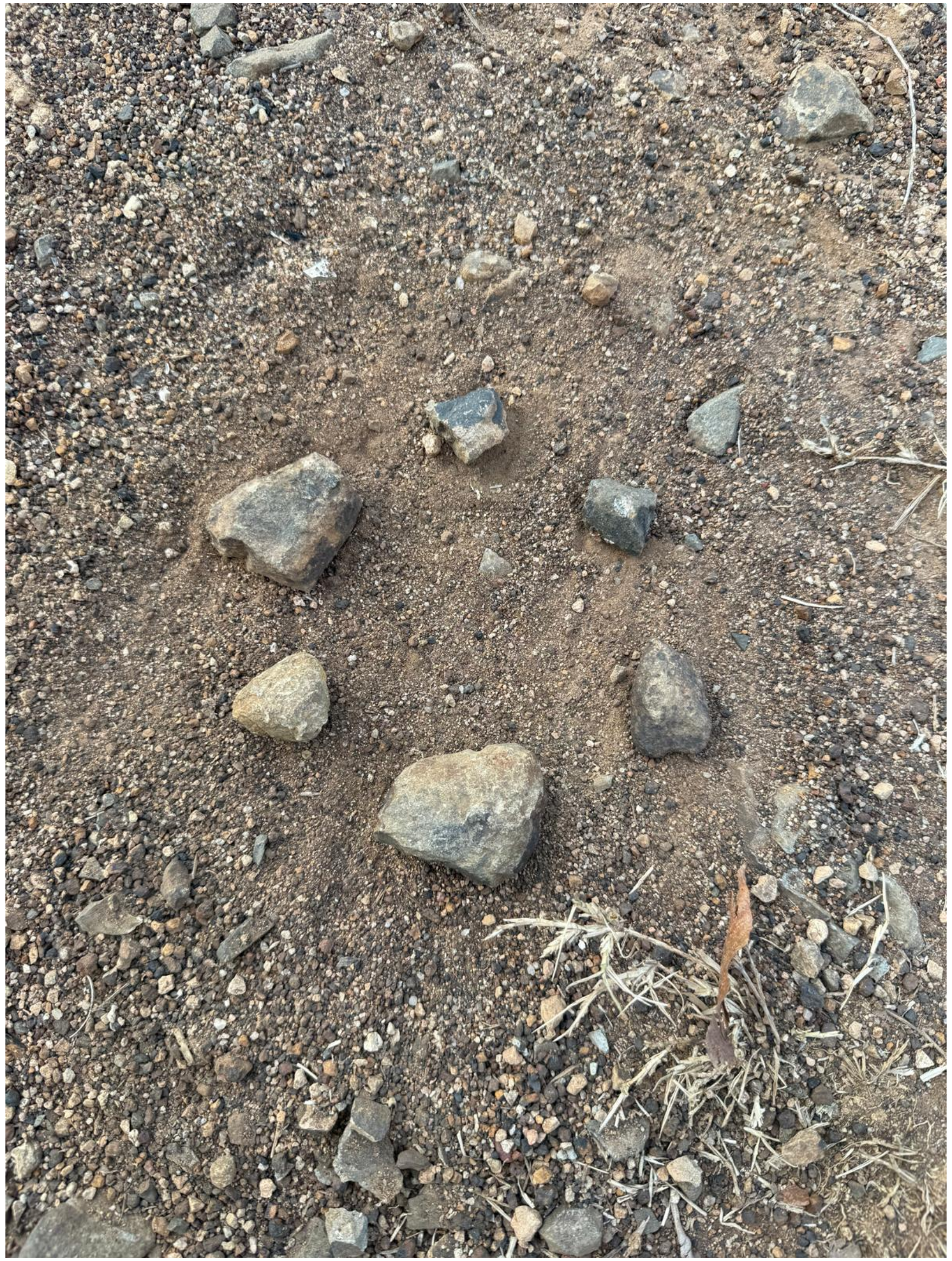

What is your task?

- What do you eat or drink? Or want to eat or drink?
- Where do you eat or drink?
- Who and when do you eat or drink?

What is your task?

- **Take pictures** with phone on some of the spaces, places, items, images, or ads that impact what you eat or want to eat.
- Take 10-20 pictures (real, symbolic, arranged).
- You decide which ones you want to show me next time we meet. You transfer the pictures to me.
- Next time we meet I will ask you to explain what the photos mean to you, and have you draw your own map of the area, and place the photos on the map.

What to think about?

- Do not take pictures of personal information (address, ID card, doctor notes, etc.)
- Do not take pictures of anyone else's face.
- Do not take pictures of any place you do not usually go to. We would like to see pictures that represent the surroundings in your daily life.
- Take photos as you walk home, or help prepare dinner, or a snack you eat during school, or during lunch

Good Luck!

Your parents can contact [redacted] in case of any questions or concerns
