# Supplementary material for: “It’s where I buy lunch or anything that I want to buy”: a participatory photo elicitation study on adolescents’ experience of food choice and environment in Kisumu, Kenya
Source: J Health Popul Nutr. 2026 Aug 24;45:190. doi: 10.1186/s41043-026-01421-8 (PMC13505041; doi:10.1186/s41043-026-01421-8)
Supplement: Supplementary file 4 [file 41043_2026_1421_MOESM4_ESM.pdf]

Article section or element	Practices to consider, congruent with Big Q approaches (some may be required by journals e. g., discussion of ethical approval)	Practices to avoid or to avoid defaulting to (without consideration and justification)	Notes for reviewers/editors less familiar with Big Q	Authors reflections on BQQRG
**Writing style**	Reflexive, subjective, personalised, humanised, contextualised, passionate (Walsh 2015), lively, evocative, compelling (Gilgun 2005) writing;The researcher written in rather than out of the methodological account (Gaddefors and Cunningham 2024).	Objective, neutral, detached, distanced, decontextualized, passive, dispassionate writing.	Big Q researchers write from a particular standpoint in a particular place and time and only can produce a partial account of their subject-matter (Walsh 2015, 43).	Attempted to write from a reflexive and contextually grounded stand point.
**Terminology**	Defining key terms and concepts (if relevant);Explaining chosen terms for any contested terminology.	Stigmatising and pathologising terminology (consult literature/communities relevant to the research area/topic).	Much terminology in qualitative research is contested. Whats important is that the researchers explain their usage rather than conforming to particular “ dominant“ understandings.	Defined terms of photo elecitation and food scape mapping along with the theoretical underpinnings of these methodologies.
**Title**	A title that captures the topic area and/or the central storyline of the analysis; potentially also the method/ology;A title that engages, intrigues, entices the reader;If aiming to maximise discoverability, a title that includes 1 to 2 keywords in first part.	Abbreviations.		Included an illustrative quote to capture adolescent perspective of emerging autonomy then followed by a more descriptive title clause.

**Abstract and keywords**	Summarising key points of the article;Contextualising the study/topic/research question;Indicating theoretical approach and study design/methodology/practices of inquiry, including participants/empirical materials;Outlining main analytic arguments/observations;Signalling conclusions/implications;Keywords that offer information beyond title, related to topic, approach, etc.3	Segmenting the abstract by the sections and content of positivist-empiricist reporting traditions (though this may be required).	Structured abstracts can limit what and how information is conveyed. Allowing flexibility to not use, or adapt, structured abstract headings helps not just to enable congruent presentation of Qualitative research, but for the essence of the research to best be conveyed.	Maintained a structured abstract approach per the journal guidelines, but outlined main thematic findings and signalled implications within the conclusion section.
**Introduction** (Authors can sequence the various elements of this section in whatever order works best for their approach/article. If a heading is needed for this section, we prefer				

**Background and rationale**	Framing the research as entering a conversation about the issue;Providing a robust context and rationale for the proposed research;Discussing (reviewing/critiquing/synthesing) existing research, theory, and the wider contexts (e.g., social, policy, practice, political, ideological, etc.) relevant to contextualising the study;Engaging deeply with any existing qualitative research;Situating other knowledge as partial and produced from a perspective (especially when discussing research with a different values base, such as quantitative) â€“ including where relevant, the implications of methodological elements of other studies.	Discussing existing quantitative literature in a way that suggests the author aligns with the philosophical assumptions underpinning it (if these are not congruent with Big Q);Centring critiquing the methodological limitations of existing research, and from a (post)positivist standpoint, as a key foundational justification for your own study (without a clear reason).	Methodological critique per se (pointing out flaws to signal how much better this study will be) is not generally a key foundational justificatory technique for Big Q; in some cases, methodological critique is appropriate (e.g., of problematic assumptions and exclusions, terminology; to make the case for a new â€“ to a topic/field â€“ methodology), but itâ€™s not a necessary element in reporting.	Provided specific contextual insights as well as wider public health implications. Rather than countering existing literature this study aims to expand upon it by centering another perspective in a different setting.

**Research question, aims, and/or purpose**	Articulating research questions, aims, and/or purpose clearly;Demonstrating methodological congruence between questions (etc.4) and overall approach;Noting if article is part of a wider study and linking to other publications, while ensuring the article focus is clear, and information provided fully informs the reader, on its own (reading other publications should only enhance understanding, not be required for it);Discussing any shift or refinement from a broader/different research question to the one addressed in the article.	Formulating research questions as hypo-theses or expectations about what might be â€˜found;â€™Implicitly formulating research questions in terms of measuring relationships between variables (e.g., effects of X on Y);In experiential qualitative, formulating research questions in ways that donâ€™t take account of subjectivity (e.g., aiming to [directly] explore X phenomenon rather than participantsâ€™ perspectives on or experiences around X phenomenon).	The practice of splitting a dataset is referred to " disparagingly" as salami slicing in quantitative research (Menon and Muraleedharan 2016). This critique sometimes gets deployed unjustly against qualitative research. It is legitimate to report a qualitative study across multiple articles; each article should have a distinct focus (Levitt et al. 2018).	The aim is clearly stated on line 103-104. Then within setting we describe how the data lies within the Changemaker project. At this point however, the data is only planned to be used for the purpose of this manuscript regarding publication.

**Owning your perspectives discussion of theory (this content may be addressed instead in the Methodology)**	Including information about guiding meta-theoretical assumptions, and any other (e.g., explanatory, political/ideological) theory informing the research;Demonstrating methodological congruence between guiding (e.g., paradigmatic, ontological and epistemological) and any other theory.	Ontological and epistemological incongruence (e.g., claiming constructionism, but focusing on lived experience and treating language as a transparent window onto this);Implicitly or explicitly using objectivist and/or foundationalist thinking, either overall or â€˜mashed-upâ€™ with Big Q assumptions (e.g., using reflexive thematic analysis [TA] but assuming data interpretation can be accurate and reliable);Using concepts and terminology incongruent with claimed theoretical framework (e.g., claiming constructionism but drawing on social cognitive constructs of body image, attitudes etc.);Treating theoretically embedded and delimited constructs as theoretically neutral/trans-theoretical (e.g., treating	The research and the report should evidence methodological congruence/integrity (Levitt et al. 2017) meaning theoretical assumptions, research questions, methodology, practices of data generation and analysis, purpose of research etc. together conceptually.Incongruence often comes in through mismatches between (stated) theoretical assumptions, and various aspects of the report, such as in the use of language and concepts (e.g., around theme development, research subjectivity, data interpretation), treatment of data, and quality practices. Common language and practices often reflect potentially conceptually incongruent (with Big Q) ideas and assumptions, so	On lines 113-119, we describe the underlying meta-theoretical assumptions of the methodology and analysis.

**Owning your perspectives discussion of reflexivity (this content may be addressed instead in the Methodology)Owning your perspectives discussion of reflexivity (this content may be addressed instead in the Methodology)(continued)**	Discussing researcher professional or personal positioning5 and experience, or the broader contexts shaping their experiences and perspectives (Lazard and McAvoy 2020), in relation to the topic and/or participant group and/or their role in shaping the research;Demonstrating critical reflexivity â€“ interrogation of social positioning and how structural and interpersonal dynamics shaped the research, and the reporting (Grzanka and Moradi 2021);Referencing reflexive journaling as part of research practice if used;Using first person writing style when discussing researcher reflexivity, decisions etc.	Referencing researcher â€˜biasâ€™ (as contamination/threat to objectivity);Referencing researcher â€˜influenceâ€™ â€“ framed as possible rather than inevitable;Referencing the researcherâ€™s prior understandings/expectations , and framing these (implicitly) as a potential source of contamination/distortion;Writing the researcher out of the research by using a third person writing style.	First person writing is good practice for Big Q, as it positions the researcher in the research (Gaddefors and Cunningham 2024).While positioning and â€˜owning perspectivesâ€™ is good practice, requiring individual researchers to disclose personal information, especially when it might be discrediting or stigmatising, and/or researchers are members of marginalised communities (Grzanka and Moradi 2021), is not.There is no ideal location for researcher reflexivity in Big Q reporting, so where reflexivity should appear in an article should not be prescriptive (Wu et al. 2016).	Included a section on reflexivity and positionality on lines 197- 208. This section serves to briefly illustrate the on going reflexive processes that the team engaged in over the course of the study.

**Methodology** (We prefer the theoretically-embedded term Methodology over the proceduralist term Method as a section title. Again, the order of information is not prescriptive, although it is typically easier to follow if rationale/approach precedes specific aspects of design and procedure. Whether and how subsection headings are used should be flexibly situationally determined flexibility helps avoid redundancy/duplication of information.)				Adapted Heading term to reflect this idea and called it Methodology.
**Research design/approach/methodology (this content may sometimes be better located earlier in the article)**	Describing the research design/approach/methodology;Providing a rationale for the selected design, etc.;Explaining the conceptual framing for any multi-method or pluralistic (vs. single method) design (e.g., Chamberlain et al. 2011).	Implicitly positioning quantitative research and designs as the norm/ideal.	Proving a default justification for qualitative research per se, as opposed to quantitative, may be required in certain contexts, but unlikely in those that welcome Big Q.	Describe the study design choosen and the rationale for it under the heading Study Design (lines 106 -208)

**Participants/data sources/empirical materials**	Describing the selection and number of participants/data items, and rationale for these;Explaining the criteria for selection/recruitment strategies;Discussing dataset size and scope with reference to non-positivist qualitative concepts, such as information power (Malterud, Siersma, and Guassora 2016) or sufficiency, orienting to the quality of the dataset to address the research aims and questions;Describing the characteristics of participants/data items6 and recruitment/data generation contexts;In research with participants, balancing the need to situate the participant group (Elliott, Fischer, and Rennie 1999) with participant anonymity (e.g., aggregating or reporting minimal demographics where appropriate);Describing the relationship between researchers and participants prior to, during, and after the research, including any ethical considerations (e.g., dual relationships, researcher as insider/outsider);Describing how the study purpose was communicated to participants, including any definitions of key terms/concepts explained;Discussing the use of pseudonyms/participant codes (including how chosen, and rationale; see	Using common terms/concepts like sample/sampling (which connotes sampling from a population for the purposes of statistical generalisation);Justifying sample size/stopping data generation with reference to criteria such as (data/code/theme) saturation (i.e., information redundancy) or statistical models as these are often not conceptually congruent (see Braun and Clarke 2021b; Varpio et al. 2017);Providing demographic information in a way that might compromise anonymity, such as a table showing demographics of each participant;Using pseudonyms or participant descriptors which compromise participant anonymity.	There is no widespread agreement on what constitutes an appropriate number of participants/data items for qualitative research, and for particular types of research question, methodologies and practices of inquiry etc.Determining a sufficient participant group/dataset size involves interpretative, situated and (especially in student and other unfunded research) pragmatic judgements.Participant group/dataset size anxiety (such as explanation for a âsmallâsample) often reflects the lingering presence of positivism; bigger is inherently better consider the quality of the data (to address the research questions/in relation to methods).As codes/numbers can dehumanise participants,	Described the recrutiment and data collection strategy on line 141-179. Utilized the concept of information power to avoid a post-postive sampling conceptualization. Further describe the relationship between participants and researchers in the positionality and reflexivity section.

**Ethical approval and ethical code/principles followedEthical approval and ethical code/principles followed(continued)**	Detailing institutional ethical approval;In research with participants, describing processes of informed consent;Detailing any incentives and compensation, and the rationale for these;Discussing any study/design/sociopolitical context etc. specific ethical considerations or practices;Discussing how any concerns related to accessibility and inclusion, equity, and intersectionality were attended to;Where relevant or appropriate, including research materials (participant information, consent form) in supplementary material.	Compromising participant anonymity by the details provided;Research is implicitly/unknowingly exclusionary (which limits the contribution of the research).	Ethical considerations in Big Q often go further, to consider issues beyond those in institutional review, so this section might be expansive.	Provided details on the consenting and assenting process at the beginning of the participants and recruitment section. Then describe the ethical approval on line 209-214.

**Data generation (we prefer the term generation over collection or gathering, as it captures the active role of the researcher and acknowledges that data donâ€™t pre-exist research as data; they become data through research)Data generation (we prefer the term generation over collection or gathering, as it captures the active role of the researcher and acknowledges that data donâ€™t pre-exist research as data; they become data through research)(continued)Data analysis procedures**	Describing and explaining practices of inquiry/data sources chosen (explains e.g., why the method/data source is a good fit with the research question, participant group, guiding theory, etc.);If relevant, explaining the purpose and use of a multi-method or pluralistic design (e.g., crystallisation, Tracy 2010);Discussing the development and characteristics of data generation tool(s)/protocol(s) and including tool(s)/protocol(s) in supplementary material where possible;Discussing â€˜pilotingâ€™ if used, and any changes to and evolution of tool(s)/protocol(s)/research design following piloting/review, and subsequently during data generation;Describing the modality (e.g., video call interviews, in person focus groups, online qualitative survey)/setting, and time frame, of data generation, and any other pertinent procedural details;Noting any audio or video recording of interactive data generation and other data â€˜recordingâ€™ practices (e.g., field notes, photography, participant/researcher drawings);Noting who (which author or role) completed any interactive data generation;Describing	Triangulation of data sources (framed in a realist/objectivist way to get closer to the truth/a more accurate understanding);Orienting to replication in how data generation is described;Treating lack of standardisation in data generation method, modality or setting as a problem, a potential source of â€˜biasâ€™ (failure of objectivity);Seeking standardisation (e.g., through the training of researchers) in interactive data generation;Equating data quantity with data quality;Transcription described as â€˜verbatimâ€™ or â€˜orthographicâ€™ with no further details provided;Simply stating transcript page numbers as a measure of the dataset with	In Big Q, research â€˜might seem delayed, late or tardy, behind where it is supposed to beâ€™ (Howell 2019, 3) â€“ for instance, reporting data generated some years past. Within a Big Q orientation, â€˜olderâ€™ data is not in and of itself a good reason for rejecting a manuscript â€“ as relevance often continues given the model is not necessarily one of new knowledge superseding old.The idea of timeliness and urgency (of data/analysis) is important to deconstruct in relation to the ways the norms and expectations of academia work against groups such as disabled academics.It is appropriate for accounts of procedures and practices to foreground some of the â€˜messâ€™ of qualitative researching: â€˜transparent research is marked by	Described the data collection in detail on lines 140 -180. We attempted to disclose various aspects of the process. The Supplemenary materials provide details on data collection tools. (We maintained data collection terminology in this instance for consistency with other literature).

**Data analysis procedures(continued)Data analysis procedures(continued)**	Describing and explaining the analytic approach;Where relevant, describing the variant of the approach and/or data orientation (e.g., inductive, deductive etc.);Describing who (author or role) analysed the data;Where analysis is collaborative, discussing how this contributed to researcher reflexivity/insight;If using an established approach, describing how the researcher(s) engaged with the analytic process, and how the analysis was developed;If tweaking/changing, or supplementing, the procedures associated with an established approach, explaining how and why;Using supplementary material, if necessary, to provide a fuller account of the analytic process;Using language appropriate to the role of the researcher in developing the analysis (e.g., in reflexive TA, language around theme development conveys the active role of the researcher in generating, crafting, constructing, creating or developing themes);Describing any software (QDAS) used during the analytic process;If tools or modalities used for analysis changed (e.g., QDAS > manual process), reflexively discussing rationale for	Citing generic characteristics of an approach (e.g., accessible, flexible) without explaining how these were relevant to the study;When using an established analytic approach, adding alternative/supplementary analytic procedures, without acknowledgement or rationale (e.g., unexplained â€˜supplementingâ€™ of the six phases of reflexive TA with the creation and use of a codebook);Using inter-coder agreement or consensus coding (as measures of coding accuracy/reliability);Simply citing established methodologies as â€˜brand namesâ€™ (Tuval-Mashiach 2017, 130), without providing further situated detail around use;Using generic descriptions of procedures or â€˜technical description of analytical phasesâ€™	One coder/analyst is entirely normal and appropriate within Big Q; it does not present a validity threat to the research.Use of QDAS is not a requirement.	The coding and analysis process is decribed in line 181 -197. The processed involved one coder and did not utilize any intercoder rating as following TA approach. Themes were considered to be generated and a product of coconstruction between the data and research team insights.

**Quality practices**	Discussing any Big Q congruent quality practices not covered elsewhere. Either here or in the discussion, reflecting on any â€˜limitationsâ€™ of the dataset/participant group, study design/approach and procedure (with reference to a contextualised Big Q orientation).	Using postpositivist criteria for quality, such as triangulation (to get closer to the truth/a more accurate understanding), analysis agreement/consensus among the research team, corroboration of analysis by another researcher;Using (a foundationalist version of) member checking/participant validation without a congruent â€“ empirical, theoretical or political â€“ rationale (see Motulsky 2021).	Congruent quality practices in Big Q can include (where appropriate, but not always): member reflections, crystallisation (Tracy 2010), other researchers serving as a critical friend/sounding board/mirror (Morrow 2005; Smith and McGannon 2017) to enhance reflexivity, reflexive journaling.	Reflected on quality practices within the strengths and limations section of the discussion. Utilized member reflections, crystallisation, and discussions with team as critical friend during analysis.

**Analysis (**For this section, we prefer the heading Analysis over Findings/Results because it connotes the presence of contestable interpretations of human phenomena (Walsh 2015, 43). In contrast, Findings infers the researcher found,discovered or identified pre-existing entities, and Results is strongly associated with the outputs of statistical analysis (Walsh 2015). Format/flow and structuring of reported analysis often varies by analytic approach.)				Maintained term Findings based on journal requirements
**Structure, number, and names of patterns/themes/categories/discourses/narratives (etc.)**	Reporting a clear analytic structure;Reporting an appropriate number of patterns/themes/categories/discourses/narratives (etc.)7 relative to the length of the article, allowing for depth and detail in the presentation of the analysis.	Overly elaborated/fragmented or unclear analytic structure;Reporting a large number of patterns (etc.) relative to the length of the article;In research using coding, reporting the number of codes (e.g., per pattern) as a validity measure.	Pattern (etc.) names can be playful, fun, provocative, and engage, entice or intrigue the reader.It s not uncommon for brief data quotations or paraphrases to be used in pattern (etc.) names usually if they capture the core of the pattern.	Organized the findings sections based on the flow of the themes pattern.

**Overview of, or introduction to, analysis, if relevant**	Including an analytic map, table or a list of patterns (etc.), or general contextualisation of the analysis;Using pattern (etc.) names that align with Analysis section subheadings.	Unexplained headings in the Analysis section (not connected to patterns [etc.] as outlined);Reported patterns (etc.) that donâ€™t match the analytic structure previewed.		Table 1 illustrated the three core themes and 8 sub themes.
**Theme/category/discourse/narrative (etc.) conceptualisation, where relevant**	Using a conceptualisation of patterns (etc.) that is congruent with the approach (e.g., themes in reflexive TA have a central organising concept and report shared meaning);Explaining/rationalising any divergences in the conceptualisation of patterns (etc.), aligned to methodological congruence.	Mismatch between pattern (etc.) conceptualisation â€“ either for methodological approach, or as described in the Methodology â€“ and what is then reported (e.g., topic summaries as themes in reflexive TA; Braun and Clarke 2022a).	Qualitative research produces many differently conceptualised analytic entities (patterns, etc.). Then within those of the same name/type, there is often considerable variation in how they are conceptualised (e.g., different ideas of â€˜a themeâ€™, â€˜discourseâ€™ etc.).	Attempted to go deeper than topic summaries and utilize descriptive themes that aim to illustrate the tension between aspects of adolescents emerging autonomy.

**Analytic depth**	Providing an analytic narrative (around data extracts) that explains the meaning and significance of the data, and researchers interpretation of them guided by principle that data do not speak for themselves;Telling a rich descriptive and/or interpretative story about the data.	Frequency counts for patterns (etc.);Analytic foreclosure (e.g., data generation questions reported as themes etc);Patterns (etc.) that are thin and one dimensional (Inayat et al. 2024);Simply paraphrasing data;Data interpretation treated as self-evident;Arguing with the data the task of Big Q analysis is generally to make sense of the data, not orient to whether its wrong or right.	Using semi-quantification (Neale, Miller, and West 2014) the use of few, some, many etc., usually without precise boundaries for these may sometimes be appropriate in describing analysis, but isnt a requirement.	Throughout the Findings sections provide narrative of the themes and do not include counts and considered any data as valuable.

**Use of data extracts, where relevantUse of data extracts, where relevant(continued)**	Using a sufficient number and range of extracts from the dataset to convincingly and compellingly evidence analytic claims;Introducing and contextualising data extracts appropriately (so readers can comprehend the content of extracts);Displaying congruence between data extracts and analytic narrative;Balancing appropriately the proportion of analytic narrative and evidentiary data extracts;Appropriately anonymising data extracts;Ensuring (meta)theoretical concepts informing the analysis, and treatment of language, are congruent with the research approach.	In approaches reporting patterning across a dataset, selecting extracts from a small proportion of the participants/data items without any acknowledgement or rationale;Providing no data extracts to evidence analytic claims, only a researcher summary;Writing one sentence/a very brief analytic narrative â€˜overviewâ€™ of the pattern (etc.), followed by several data extracts without further analytic elaboration. Without adequate analytic narrative, the reader ends up â€˜drowning in a sea of endless dataâ€™ (Chenail 1995, 7);Insufficient contextualisation of data extracts making the (literal) meaning unclear;Mismatch between analytic claims and data extracts presented to evidence those claims (analysis is not	Further data extracts and analysis can be included in supplementary materials (Levitt et al. 2017).The appropriate balance of analytic narrative and data extracts will depend on the analytic method/ology used and the particular orientation (e.g., inductive/deductive, experiential/critical, descriptive/interpretative);Data extracts may be used illustratively, as general instances of analytic claims (readily interchangeable with other examples without disrupting the analytic narrative). They can also be used analytically, where the analysis is developed through commentary on specific features of a data extract. An analytic use typically involves fewer data extracts, and more analytic narrative.	Utilize quotes, pictures, and the maps as illustrative tools to exemplify the themes and expand the narrative.

**Integration of analysis with existing research and theoryIntegration of analysis with existing research and theory(continued)**	Drawing on existing research and theory to contextualise analysis and (potentially) to deepen data interpretation â€“ here theory is not primarily about conceptual or metatheory (ontology/epistemology etc.) so much as the explanatory frameworks and concepts that help interpret the data (e.g., healthism; resilience; consent);Weaving such research and theory through the analytic narrative, as relevant.	Separation of data/Results and interpretation;Description/summary rather than interpretation of data.	Integration of other literature into the Analysis section is common in Big Q: the analysis section is not a results section that describes findings without commentary, as you might find in the write-up of an experimental study (Riley 2023, 220).The literature discussed in the Analysis doesn't have to be confined to that discussed in the Introduction.	The theory was interweaved in the analysis process, however, we did contain explicit contextualization within other literature and the theoretical framework to the discussion.
**Discussion/Conclusion**The scope of the Discussion/Conclusion will depend on how integrated results and discussion were in the previous Analysis section. We recommend avoiding repetition of earlier content in this section, except in the service of developing the arguments of the article further.				

**Analytic conclusions and contributions**	Drawing analytic conclusions across patterns (etc.);Discussing the contributions the research has made to existing research, theory, etc, oriented to the broader context of the study, both scholarly and societally;Considering how our knowledge has shifted what/how we know about the topic of the research (related to research).	Repetitive pattern-by-pattern (etc.) integration of the analysis with existing literature, rather than telling an overall analytic story or drawing overall conclusions.	The literature drawn on doesnt have to be confined to that discussed in the introduction, and often isnt.	Recontextualized the themes within the Sobal framework and integrated with other literature non introduced earlier.
**Study evaluation/reflectionStudy evaluation/reflection(continued)**	Overall reflexive consideration of the context, process, outcomes and potential consequences of the research. Reflecting openly/honestly on strengths and limitations of the study design, practices of inquiry, procedures, researcherâ€™s decisions, etc., oriented to the scope and parameters of the research itself;If no separate discussion of researcher reflexivity in this section, reflecting on the researcherâ€™s role in shaping the research and the knowledge generated;If relevant, considering what other researchers, such as those working with the same participant community, using the same practices of inquiry, etc., might learn from your study (both the doing of the study, and the analysis produced);Using qualitative conceptions of generalisability (e.g., Smith 2018).	Orienting to dominant quantitative norms in evaluation, such as describing the â€˜sampleâ€™ as â€˜smallâ€™, or â€˜nonrepresentativeâ€™;Lack of generalisability framed as a weakness through implicitly conceptualising this concept as statistical generalisability;Researcher bias/influence (as contamination/threat to objectivity) noted as a limitation.	From a Big Q standpoint, a study evaluation/reflection might include consideration of:The characteristics and context of the participant group/dataset;How particular methods and modalities shaped the data generated;How the researcher(s) shaped the data generated;Potential â€˜transferableâ€™ implications of the research.	Include a strengths and limitation section to describe the quality of knowledge generated on line 444 - 458.

**Researcher reflexivity**	Reflecting on the researcherâ€™s role in the research process and practices, including (further) researcher reflexivity, where relevant.	Providing a â€˜laundry listâ€™ of identity positions, framed as influencing the research;Evoking the notion of researcher bias/influence (as contamination/threat to objectivity) â€“ including through idea that researcher may have influenced the research.	Within Big Q, researcher â€˜influenceâ€™ is understood as inherent â€“ so itâ€™s a question of how the research was â€˜influenced,â€™ not whether. Using â€˜ifâ€™ instead of â€˜howâ€™ to discuss influence suggests separation is possible (and perhaps ideal).	Included in the methodology section
**Implications for future research, policy and practice, where relevant**	Making evidence-based (where available) suggestions for future research (e.g., provide grounds for other groups potentially having different experiences/views);Discussing implications for practice and â€˜actionableâ€™ outcomes (Sandelowski and Leeman 2012).	Providing a â€˜laundry listâ€™ of other groups/communities to research around this issue.	Since complete-population knowledge and/or uncontextualized generalisability are not the aim in Big Q research, it does not make (inherent) sense to suggest researching the topic within another community/group.	Included a section on implications for practice and research to describe the potential uses of the analysis within this population (line 427-443).
